# Inhibition of HSC70 alleviates hypertrophic cardiomyopathy pathology in human induced pluripotent stem cell‐derived cardiomyocytes with a MYBPC3 mutation

**DOI:** 10.1002/ctm2.647

**Published:** 2021-12-29

**Authors:** Hangyuan Qiu, Yaxun Sun, Ziwei Pan, Jingjun Zhou, Hongkun Wang, Xiaochen Wang, Dongsheng Cai, Guosheng Fu, Tingyu Gong, Chenyang Jiang, Ping Liang

**Affiliations:** ^1^ Department of Cardiology Sir Run Run Shaw Hospital Zhejiang University School of Medicine Hangzhou China; ^2^ Key Laboratory of Combined Multi‐Organ Transplantation Ministry of Public Health The First Affiliated Hospital Zhejiang University School of Medicine Hangzhou China; ^3^ Institute of Translational Medicine Zhejiang University Hangzhou China

Dear Editor,

We performed a comprehensive study to assess the pathogenicity of a cardiac myosin binding protein C3 (MYBPC3) variant (L460fs) and to pinpoint underlying molecular mechanisms by utilizing human‐induced pluripotent stem cell‐derived cardiomyocyte (iPSC‐CM) model. L460fs iPSC‐CMs exhibited a variety of deleterious phenotypes in response to angiotensin II (Ang II), including reduced MYBPC3 expression, hypertrophy, arrhythmia and elevated diastolic intracellular Ca^2+^ [Ca^2+^]_i_. Mechanistically, heat shock protein family A (HSP70) member 8 (HSC70) accelerated MYBPC3 degradation via lysosomal pathway under Ang II stress. The reduced MYBPC3‐binding ryanodine receptor 2 (RYR2) caused by insufficiency of MYBPC3 protein may give rise to excessive free destabilized RYR2, which in turn promoted RYR2‐mediated Ca^2+^ leak. The resultant elevated Ca^2+^ loading may trigger the development of both hypertrophy and arrhythmogenesis, particularly under stress conditions.^1^

Hypertrophic cardiomyopathy (HCM), featured by asymmetric ventricular hypertrophy, arrhythmias and sudden cardiac death (SCD), is the most common form of inherited cardiac disease.^2^, ^3^ To note, HCM has been repeatedly regarded as a major cause of SCD in young people.^4^ *MYBPC3*, which encodes cardiac myosin‐binding protein C, is the most frequent HCM‐associated gene, accounting for more than 50% of the HCM patients.^5^ However, the exact mechanism of MYBPC3‐related HCM remains to be resolved.^6^, ^7^

In this study, a MYBPC3 variant (c.1377delC; p.L460fs) was identified in four‐unrelated individuals who developed HCM in middle age (Figure 1A,B). The echocardiography and cardiac magnetic resonance imaging exhibited normal ventricular function, severe interventricular septum hypertrophy but without left ventricular outflow tract obstruction (Figure 1C, Figure S1A‐D and Table S1). With treatment of β blockers or Ca^2+^ channel blockers, the symptoms were significantly relieved. Family history of SCD was presented in family 1 and 4 (Figure 1A). For proband 1, implantable cardioverter defibrillator (ICD) was implanted to prevent SCD, and radiofrequency ablation was applied to eliminate atrial fibrillation. Proband 4 suffered a lethal ventricular fibrillation before ICD implantation with much more diffused late gadolinium enhancement and frequent non‐sustained ventricular tarchycadia (VT) compared to others (Figure 1C,D). Trans‐epicardial ablation was applied to relief the burden of VT. As for proband 2 and 3, only mild symptom was presented and medical therapy was sufficient (Figure S1E and Table S1).

**FIGURE 1:**
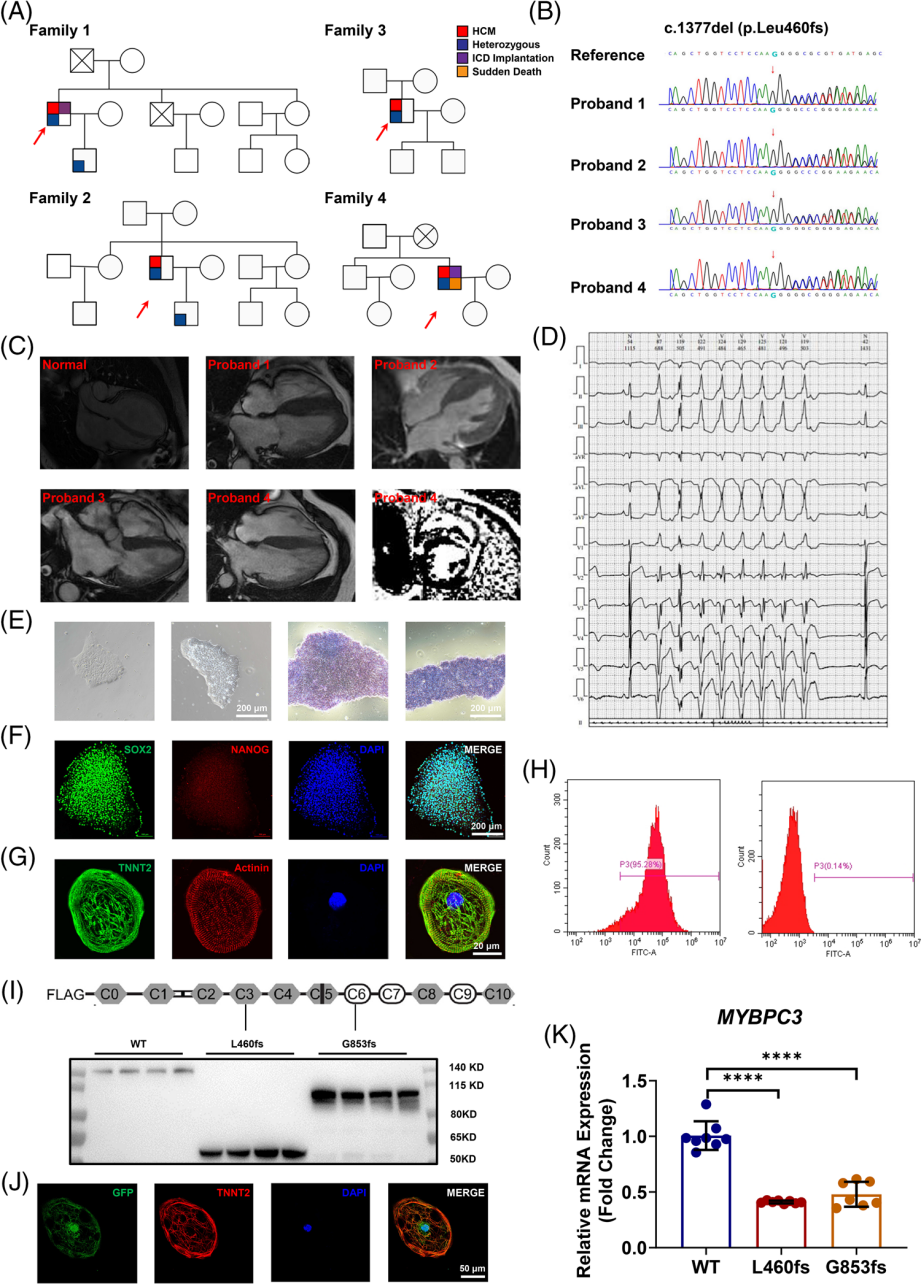
Identification of a myosin binding protein C3 (MYBPC3) mutation in four hypertrophic cardiomyopathy (HCM) probands and overexpression of the MYBPC3 truncation mutations in healthy induced pluripotent stem cell‐derived cardiomyocytes (iPSC‐CMs). (A) Pedigree of four HCM‐affected families carrying a MYBPC3 mutation (c.1377del; p.L460fs). Individuals who were dead are marked with a fork. The probands are indicated by red arrow. Since June 2019, 374 HCM patients who presented with significant left ventricular (LV) hypertrophy and typical symptoms such as palpitation, angina and syncope have been enrolled in our study. In family 1, the brother of index patient died suddenly. The index patient developed a paroxysmal atrial fibrillation (AF), and radiofrequency ablation was applied to restore sinus rhythm. Implantable cardioverter defibrillator (ICD) was also implanted for sudden cardiac death (SCD) prevention. After procedure, amiodaron and β‐blocker were administrated, and no AF‐like event has happened. The son of index patient carried the same MYBPC3 mutation but present no symptom. The fourth probands experienced a lethal ventricular fibrillation at age of 56. Typical characterization of HCM was presented in echocardiography and cardiac magnetic resonance imaging. Much more diffused late gadolinium enhancement and more frequent non‐sustained VT was observed compared to other probands. Besides ICD implantation, a trans‐epicardial VT ablation was applied. The radiofrequency energy was delivered to isolate the scar. Since then, the patient has recovered well without ICD discharge. The Whole‐exome sequencing (WES) of his brother and daughter revealed no mutation of MYBPC3. For family 2 and 3, symptom was slight and only presented in index patients. None of the family members reported an unexpected sudden death. In these four HCM families, there is no hint for an early manifestation of HCM but seems to have a higher risk of SCD. (B) Representative Sanger sequencing results of the MYBPC3 locus in four different HCM probands with the red arrow indicating the mutation. WES was performed in each patient and their relatives to discover the causative gene(s). Among all 374 patients, a single nucleoid deletion in MYBPC3 (c.1377del; p.L460fs) was presented in four separated probands and their kindreds described as 'pathogenic' by the ClinVar database. (C) Representative long‐axis MRI images of four probands and a control at end diastole demonstrating asymmetric hypertrophy of the septum. A significant late gadolinium enhancement, mainly appeared in the basal hypertrophied septum, can be observed in proband 4. (D) 24‐h Holter suggests multiple events of short bursts of ventricular tachycardia (VT) in proband 4. (E) Typical morphology and alkaline phosphatase (ALP) staining of healthy induced pluripotent stem cell‐derived. Scale bar, 200 μm. (F) Representative graphs of pluripotent staining of healthy iPSCs using SOX2 (green) and Nanog homeobox (NANOG) (red). DAPI (4′,6‐Diamidine‐2′‐phenylindole dihydrochloride) indicates nuclear staining (blue). Scale bar, 200 μm. The healthy iPSCs exhibited characteristic human embryonic stem cell‐like morphology, displayed ALP activity, and stained positively for the pluripotency markers SOX2 and NANOG. (G) Representative graphs of Troponin T2, cardiac type (TNNT2) (green) and α‐actinin (red) staining of healthy iPSC‐CMs. DAPI indicates nuclear staining (blue). Scale bar, 20 μm. A small molecule‐based 2D monolayer cardiac differentiation protocol was applied. Around day 10 after the cardiac differentiation, differentiated cells demonstrated cardiac morphology with spontaneous beating and positive staining of cardiac‐specific markers TNNT2 and α‐actinin. (H) Fluorescence‐activated cell sorting (FACS) analysis presents a purification of iPSC‐CMs >95%. After cardiac differentiation, the iPSC‐CMs were subsequently experienced with non‐glucose treatment for 3 days, which can give rise to a purification of >95% through FACS analysis of TNNT2‐positive cells. (I) MYBPC3 protein schematic showing protein domains and locations of mutations. Representative Western blot of wile type (WT) and truncated (p.L460fs, p.G853fs) MYBPC3 overexpressed in human embryonic kidney 293T (HEK293T) cells. Both mutations resulted in truncated proteins that can be observed using an antibody detecting N‐terminus of MYBPC3 in all isogenic comparisons. (J) Representative graphs of green fluorescent protein (GFP) (green) and TNNT2 (red) staining of iPSC‐CMs after lentivirus introduction. DAPI indicates nuclear staining (blue). Scale bar, 50 μm. WT MYBPC3 (WT), MYBPC3 p.L460fs (L460fs) and MYBPC3 p.G853fs (G853fs) were in parallel overexpressed in healthy iPSC‐CMs by lentivirus for 24 h, and a significant GFP fluorescence can be observed under fluorescent microscope. (K) Bar graph to compare the mRNA expression of *MYBPC3* between WT and mutant iPSC‐CMs. *n* = 7–8 technical replicates. ^****^ *p *< .0001. Three days after transduction, total mRNA expression of *MYBPC3* was detected. Total *MYBPC3* mRNA expression were reduced by 59% and 53% in L460fs and G853fs iPSC‐CMs, when compared to WT counterparts

To characterize the *MYBPC3* variant in vitro, we created human WT and L460fs MYBPC3 constructs, which were overexpressed in healthy iPSC‐CMs (Figure 1E‐J). A known mutation (G853fs), previously identified as pathogenic in HCM patients, was utilized as a positive control.^8^ Both mutations are nonsense, resulting in truncated proteins that can be observed when overexpressed in human embryonic kidney 293T cells (Figure 1I). Total mRNA level of MYBPC3 was significantly decreased in both mutant iPSC‐CMs as compared to WT (Figure 1K and Table S2).

Baseline characteristics of WT and mutant iPSC‐CMs were comparable (Figure 2 and Figures S2‐S3). However, Ang II‐treated mutant iPSC‐CMs showed clear hypertrophy phenotype, including enlarged cell size and significant up‐regulation of hypertrophic marker expression (Figure 2A‐F and Figures S2‐S3). Along with the hypertrophy phenotype, MYBPC3 protein expression was significantly decreased in Ang II‐treated mutant iPSC‐CMs (Figure 2G‐H). Moreover, fura‐2 ratiometric Ca^2+^ imaging demonstrated a predisposition of arrhythmia‐like Ca^2+^ transients and higher level of diastolic [Ca^2+^]_i_ in Ang II‐treated mutant iPSC‐CMs (Figure 2I‐K, Figure S4A‐D and Table S3). It has been reported that MYBPC3 may bind to RYR2 to form a complex for stabilizing RYR2‐dependent Ca^2+^ release.^9^ Notably, we found that largely enhanced RYR2‐mediated Ca^2+^ leak contributed to Ca^2+^ handling abnormalities in Ang II‐treated mutant iPSC‐CMs, whereas sarcoplasmic reticulum (SR) Ca^2+^ load was unchanged (Figure 2L‐N, Figure S4E and Table S4).

**FIGURE 2:**
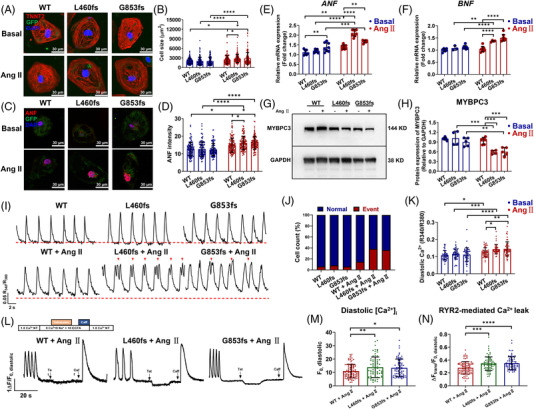
Induced pluripotent stem cell‐derived cardiomyocyte (iPSC‐CM) overexpressing myosin binding protein C3 (MYBPC3) truncation mutations exhibit Ang II‐induced hypertrophy phenotype, reduced MYBPC3 protein expression and Ca^2+^ handling abnormalities. (A) Representative graphs of cardiac‐specific staining of GFP (green) and TNNT2 (red) in basal WT iPSC‐CMs (WT Basal), Ang II‐treated WT iPSC‐CMs (WT Ang II), basal mutant iPSC‐CMs (L460fs or G853fs Basal) and Ang II‐treated mutant iPSC‐CMs (L460fs or G853fs Ang II). DAPI indicates nuclear staining (blue). Scale bar, 30 μm. (B) Bar graph to compare the cell size between different groups. *n* = 148–150 cells. ^*^ *p *< .05 and ^****^ *p *< .0001. After treatment of 5 μM Ang II for 48 h, both L460fs and G853fs iPSC‐CMs displayed a significantly enlarged cell size compared to WT (WT: 2433 μm^2^ ± 1321; L460fs: 2818 μm^2^ ± 1288; G853fs: 2858 μm^2^ ± 1859). (C) Representative graphs of staining of GFP (green) and hypertrophic‐specific marker atrial natriuretic factor (ANF) (red) in different groups. DAPI indicates nuclear staining (blue). Scale bar, 30 μm. (D) Bar graph to quantify the ANF intensity between different groups. *n* = 115–117 cells. ^*^ *p *< .05 and ^****^ *p *< 0.0001. Ang II‐treated mutant iPSC‐CMs showed intense signals of ANF, when compared to WT cells (WT: 14.9 ± 4.00; L460fs: 16.2 ± 3.85; G853fs: 16.3 ± 3.67). (E and F) Bar graphs to compare the mRNA expression of *ANF* and *BNF* by qPCR between different groups. *n* = 3–8 technical replicates. ^**^ *p *< .01, ^***^ *p *< 0.001 and ^****^ *p *< .0001. (G) Western blot analysis of MYBPC3 expression in WT and mutant iPSC‐CMs with or without Ang II treatment. GAPDH (glyceraldehyde‐3‐phosphate dehydrogenase) is used for the loading control. (H) Bar graph to compare the MYBPC3 expression between different groups. *n* = 4–6 culture replicates. ^**^ *p *< .01 and ^***^ *p *< .001. No truncated peptide was detected, and full‐length MYBPC3 protein level was compensated in both WT and mutant iPSC‐CMs at baseline by Western blot analysis. In contrast, protein levels of MYBPC3 were reduced by 37% and 36% in Ang II‐treated L460fs and G853fs iPSC‐CMs as compared to their WT counterparts. (I) Representative Ca^2+^ transient tracings recorded from WT and mutant iPSC‐CMs with or without Ang II treatment. Arrhythmia‐like abnormal Ca^2+^ transient events are indicated by red arrows. Red dash lines indicate .1 R_340_/R_380_. (J and K) Bar graphs to compare the percentage of cells exhibiting abnormal Ca^2+^ transient events and diastolic [Ca^2+^]_i_ between different groups. *n* = 47–51 cells. ^*^ *p *< .05, ^**^ *p *< .01, ^***^ *p *< .001 and ^****^ *p *< .0001. After Ang II treatment, mutant iPSC‐CMs exhibited a predisposition of arrhythmia‐like Ca^2+^ transients (WT: 17.5%; L460fs: 62.1%; G853fs: 56.7%), and a significantly higher level of diastolic [Ca^2+^]_i_, when compared to WT. (L) Representative traces of cytosolic Ca^2+^ fluorescence in WT and mutant iPSC‐CMs in NT solution and exposed to 0 Na^+^, 0 Ca^2+^ solution containing tetracaine (Tet) and caffeine (Caff). To elucidate the source of elevated diastolic [Ca^2+^]_i_, ryanodine receptor 2 (RYR2)‐mediated Ca^2+^ leak and sarcoplasmic reticulum (SR) Ca^2+^ load were assessed in iPSC‐CMs upon Ang II induction. Cells were placed in normal Tyrode's solution (NT) solution for at least 15 s before being switched to 0 Na^+^, 0 Ca^2+^ Tyrode's solution. Addition of tetracaine (1 mM), an RYR2 inhibitor that occasions a global Ca^2+^ shift from the cytosol to the SR, caused a drop in diastolic Ca^2+^ fluorescence, the magnitude of which is an estimate of RYR2‐mediated Ca^2+^ leak. Addition of a high‐concentration caffeine (10 mM), which evokes an exhaustive release of Ca^2+^ from the SR into the cytosol, induced a dramatic rise in Ca^2+^ fluorescence, an estimate of SR Ca^2+^ load. (M and N) Bar graphs to compare the diastolic [Ca^2+^]_i_ and RYR2‐mediated Ca^2+^ leak between different groups. *n* = 74–93 cells. ^*^ *p *< .05, ^**^ *p *< .01, ^***^ *p *< .001 and ^****^ *p *< .0001

We next performed genome‐wide RNA sequencing by comparing Ang II‐treated WT and L460fs iPSC‐CMs (Figure 3A and Figure S5). A significant enrichment of pathways involved in cardiac hypertrophy and cardiac muscle contraction was detected, suggesting a common gene signature of HCM in Ang II‐treated L460fs iPSC‐CMs (Figure 3B‐C). Interestingly, heat shock cognate 70 kDa (HSC70) expression was significantly up‐regulated in Ang II‐treated L460fs iPSC‐CMs at both mRNA and protein levels (Figure 3D‐G and Figure S6A‐C). To investigate if changes of HSC70 expression may give rise to functional consequences, either HSC70 activator (YM‐1) or inhibitor (VER‐155008) was applied to WT and mutant iPSC‐CMs. Addition to YM‐1 in mutant iPSC‐CMs caused hypertrophic response whereas VER‐155008 treatment or HSC70 knockdown in Ang II‐treated mutant iPSC‐CMs effectively rescued the hypertrophy phenotype (Figure 3H‐K, Figure S6D‐G and Figure S7). Nuclear translocation of nuclear factor of activated T cells triggered by Ang II was significantly inhibited by VER‐155008 treatment (Figure S8). Moreover, elevated diastolic [Ca^2+^]_i_ and enhanced RYR2‐mediated Ca^2+^ leak in Ang II‐treated mutant iPSC‐CMs were markedly restored by VER‐155008 treatment (Figure 3L‐O and Table S4).

**FIGURE 3:**
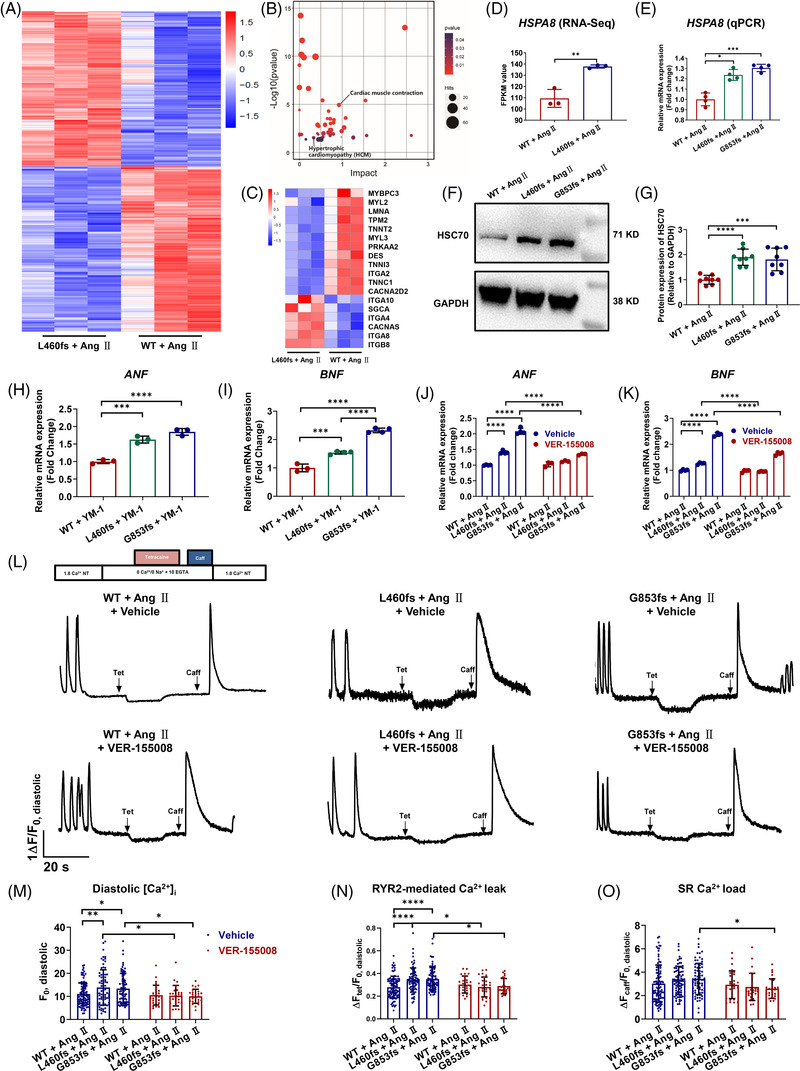
Inhibition of HSC70 rescues hypertrophy and Ca^2+^ handling abnormalities in Ang II‐treated induced pluripotent stem cell‐derived cardiomyocyte (iPSC‐CM) overexpressing myosin binding protein C3 (MYBPC3) truncation mutations. (A) Differentially expressed genes (DEGs) between Ang II‐treated WT and L460fs iPSC‐CMs were shown in heat map. (B) Kyoto Encyclopedia of Genes and Genomes (KEGG) pathway analysis identified 47‐related pathways including hypertrophic cardiomyopathy (HCM) and cardiac muscle contraction. (C) Heat map to show selected genes from KEGG analysis related to HCM. (D and E) Bar graphs to show significant upregulation of *HSPA8* mRNA expression in Ang II‐treated L460fs iPSC‐CMs by both RNA‐Seq and qPCR. *n* = 3–5 technical replicates. ^*^ *p *< .05, ^**^ *p *< .01 and ^***^ *p *< .001. (F) Western blot analysis of HSC70 expression in WT and L460fs iPSC‐CMs after Ang II treatment. GAPDH is used for the loading control. (G) Bar graph to compare the HSC70 expression between different groups. *n* = 8 technical replicates. ^***^ *p *< .001 and ^****^ *p *< .0001. (H and I) Bar graphs to compare the mRNA expression of *ANF* and *BNF* by qPCR between WT and mutant iPSC‐CMs after YM‐1 treatment. *n* = 3–4 technical replicates. ^***^ *p *< .001 and ^****^ *p *< .0001. Addition of YM‐1 (10 μM, 12 h) in mutant iPSC‐CMs significantly increased the mRNA expression levels of *ANF* and *BNF*, when compared to WT iPSC‐CMs. (J and K) Bar graphs to compare the mRNA expression of *ANF* and *BNF* by qPCR between WT and mutant iPSC‐CMs after VER‐155008 treatment. *n* = 3–4 technical replicates. ^**^ *p *< .01, ^***^ *p *< .001 and ^****^ *p *< .0001. Treatment of VER‐155008 (10 μM, 48 h) in Ang II‐treated mutant iPSC‐CMs effectively rescued the upregulated mRNA expression of *ANF* and *BNF*. (L) Representative traces of cytosolic Ca^2+^ fluorescence in WT and mutant in NT solution and exposed to 0 Na^+^, 0 Ca^2+^ solution containing Tet and Caff after VER‐155008 treatment. (M and O) Bar graphs to compare the diastolic [Ca^2+^]_i_, RYR2‐mediated Ca^2+^ leak, and SR Ca^2+^ load between different groups. *n* = 26–93 cells. ^*^ *p *< .05 and ^**^ *p *< .01

It has been previously shown that HSC70 interacted with MYBPC3 and participated in its degradation.^10^ We thus reasoned that up‐regulated HSC70 may accelerate MYBPC3 degradation. When applied to YM‐1, mutant iPSC‐CMs showed significantly reduced MYBPC3 protein expression in comparison with WT (Figure 4A,B). However, MYBPC3 protein expression in Ang II‐treated mutant iPSC‐CMs was markedly restored by VER‐155008 treatment (Figure 4C,D). We further assessed the effect of HSC70 on MYBPC3 degradation in healthy iPSC‐CMs. After cycloheximide (CHX) treatment, protein expression level of MYBPC3 in healthy iPSC‐CMs was dramatically reduced at baseline, which can be partially rescued by HSC70 inhibition (Figure 4E and Figure S9A). Notably, treatment of chloroquine (CQ) but not MG‐132 greatly rescued the CHX‐induced MYBPC3 degradation in healthy iPSC‐CMs (Figure 4F and Figure S9B). Collectively, these results suggest that increased HSC70 accelerates MYBPC3 degradation via lysosomal pathway in response to Ang II, while HSC70 inhibition restores MYBPC3 expression, thus alleviating hypertrophy phenotype and Ca^2+^ mishandling in mutant iPSC‐CMs (Figure 4G).

**FIGURE 4:**
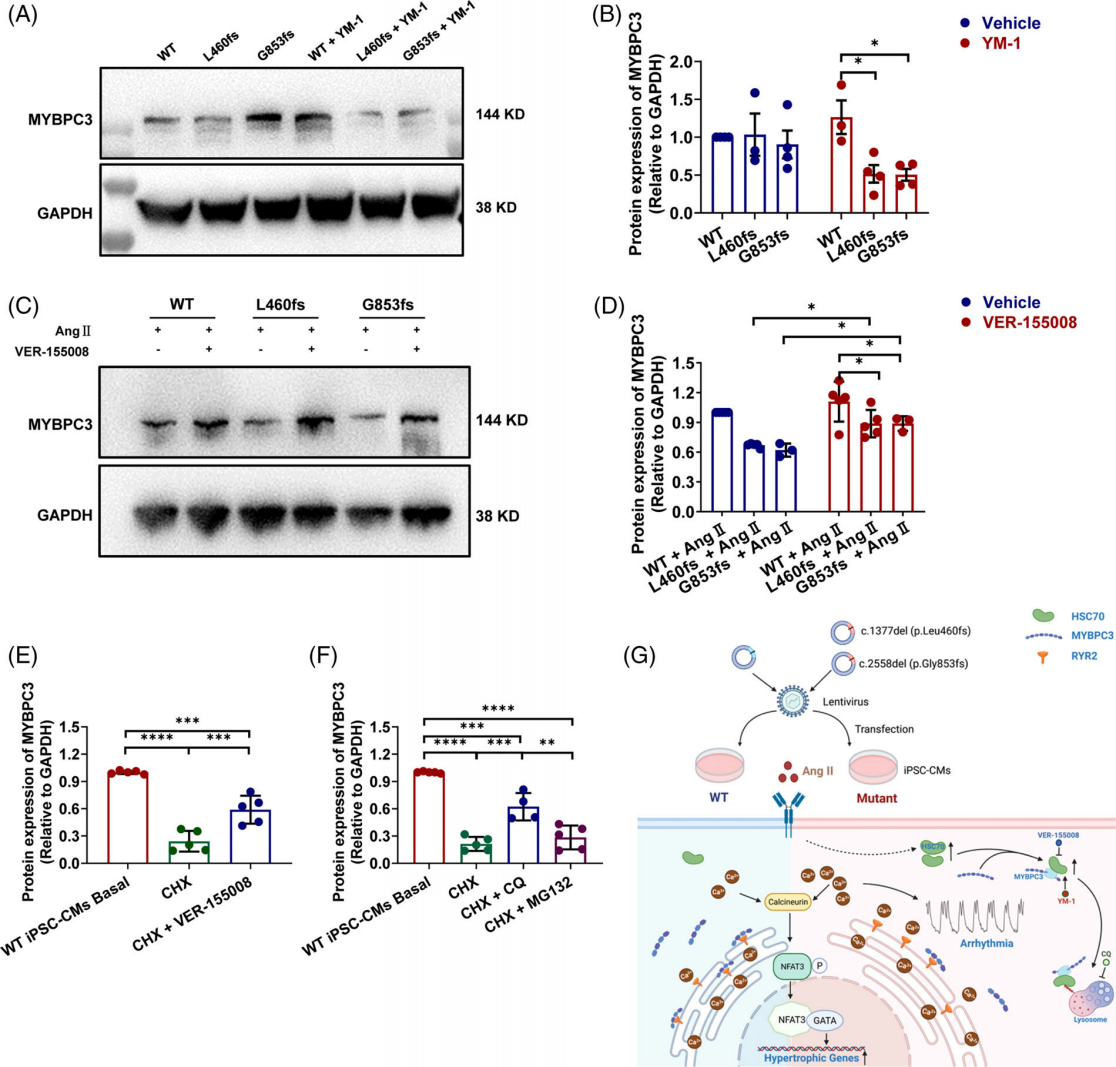
HSC70 accelerates myosin binding protein C3 (MYBPC3) protein degradation via lysosomal pathway in Ang II‐treated induced pluripotent stem cell‐derived cardiomyocyte (iPSC‐CM) overexpressing MYBPC3 truncation mutations. (A) Western blot analysis of MYBPC3 expression in WT and mutant iPSC‐CMs after YM‐1 treatment. GAPDH is used for the loading control. (B) Bar graph to compare the MYBPC3 expression between different groups in A. *n* = 3–4 culture replicates. ^*^ *p *< 0.05. (C) Western blot analysis of MYBPC3 expression in WT and mutant iPSC‐CMs after VER‐155008 treatment. GAPDH is used for the loading control. (D) Bar graph to compare the MYBPC3 expression among different groups in C. *n* = 3‐5 culture replicates. ^*^ *p *< .05. (E) Bar graph to compare the MYBPC3 expression between different groups in Figure S9A. *n* = 3–5 culture replicates. ^***^ *p *< .001 and ^****^ *p *< .0001. (F) Bar graph to compare the MYBPC3 expression between different groups in Figure S9B. *n* = 4–5 culture replicates. ^**^ *p *< .01, ^***^ *p *< .001 and ^****^ *p *< .0001. (G) Proposed work model. Ang II stress in iPSC‐CMs overexpressing MYBPC3 truncation mutations (L460fs and G853fs) causes a variety of deleterious phenotypes, including reduced MYBPC3 expression, hypertrophy, arrhythmia and elevated diastolic [Ca^2+^]_i_. HSC70 plays a pivotal role in the turnover of MYBPC3 via lysosomal pathway, which can be inhibited by chloroquine (CQ). The manipulation of HSC70 activity by YM‐1 (HSC70 activator) or VER‐155008 (HSC70 inhibitor) can regulate the content of MYBPC3 protein. In Ang II‐treated mutant iPSC‐CMs, the up‐regulated HSC70 accelerates the MYBPC3 degradation and results in the deficiency of MYBPC3 protein, which can be partially rescued by VER‐155008. The reduced MYBPC3‐binding ryanodine receptor 2 (RYR2) caused by insufficiency of MYBPC3 protein may give rise to excessive free destabilized RYR2, which in turn causes larger RYR2‐mediated Ca^2+^ leak. The resultant elevated Ca^2+^ loading may trigger the development of both hypertrophy and arrhythmogenesis, particularly under stress conditions

In conclusion, we present accelerated HSC70‐mediated MYBPC3 protein degradation as a novel mechanism of enhanced diastolic Ca^2+^ leak from RYR2 in HCM, leading to hypertrophy and aberrant Ca^2+^ handling. Our findings will be helpful for elucidating the molecular mechanisms underlying MYBPC3‐related HCM and for identifying novel therapeutic drugs for the disease.

## FUNDING INFORMATION

National Key R&D Program of China, Grant Number: 2017YFA0103700; National Natural Science Foundation of China, Grant Numbers: 81922006, 81870175 and 81970269; Natural Science Foundation of Zhejiang Province, Grant Number: LD21H020001; National Natural Science Foundation of China, Grant Number: 81970269; Key Research and Development Program of Zhejiang Province, Grant Number: 2019C03022.

## CONFLICT OF INTEREST

The authors declare that they have no conflict of interest.

## Supporting information



Supporting information
