# Supplementary material for: Inhibition of HSC70 alleviates hypertrophic cardiomyopathy pathology in human induced pluripotent stem cell‐derived cardiomyocytes with a MYBPC3 mutation
Source: Clin Transl Med. 2021 Dec 29;11(12):e647. doi: 10.1002/ctm2.647 (PMC8715830; doi:10.1002/ctm2.647)
Supplement: Supplementary file 1 — Supporting information [file CTM2-11-e647-s001.pdf]

SUPPLEMENTAL METHODS

Culture and maintenance of iPSCs

iPSCs were cultured in feeder-free mTeSR (STEMCELL Technologies) media on matrigel-coated (Corning) plates at 37°C with 5% (vol/vol) CO_2. The media were daily changed and cells were passaged every 3-4 days using Accutase (STEMCELL Technologies).

Alkaline phosphatase staining

Alkaline phosphatase (ALP) staining was performed using the VECTOR Blue Alkaline Phosphatase Substrate Kit (Vector Laboratories) following the manufacturer's instructions.

Cardiac differentiation

The iPSC-CMs were generated using a 2D monolayer differentiation protocol as previously described [28, 29]. Briefly, ~10^5 undifferentiated cells were dissociated and re-plated into matrigel-coated 6-well plates. Cells were cultured and expanded to 85% cell confluence, and then treated for 2 days with 6 μM CHIR99021 (Axon Medchem) in RPMI and B27 supplement minus insulin (RPMI+B27-Insulin) (Gibco) to activate Wnt signaling pathway. On day 2, cells were placed in RPMI+B27-Insulin with CHIR99021 removal. On days 3-4, cells were treated with 5 μM IWR-1 (Sigma-Aldrich) to inhibit Wnt signaling pathway. On days 5-6, cells were removed from IWR-1 treatment and placed in RPMI+B27-Insulin. From day 7 onwards, cells were placed and cultured in RPMI and B27 supplement with insulin (RPMI+B27+Insulin) (Gibco) until beating was observed. Cells were glucose-starved

for 3 days with RPMI+B27+Insulin for the purification. Cardiomyocytes of day 30-40 after cardiac differentiation were utilized for downstream functional assays.

Fluorescence-activated cell sorting (FACS) analysis of iPSC-CMs

Monolayer cardiomyocytes were dissociated into single cells using 0.25% Tripsin-EDTA (Gibco) for 5 minutes at 37°C. Cells were pelleted and fixed with 4% Paraformaldehyde (PFA) (Sangon Biotech) for 10 minutes on ice. Every step was washed with Dulbecco's phosphate buffered saline (DPBS) (Sangon Biotech) before sample centrifugation. Cells were stained with TNNT2 (Abcam, ab45932, 1:400) at 4°C and FITC-conjugated goat anti-mouse IgG antibody (Invitrogen) was used as secondary antibody.

Overexpression of MYBPC3 in iPSC-CMs

MYBPC3 mutants were generated by site-directed mutagenesis using the Mut Express II Fast Mutagenesis Kit V2 (Vazyme) from WT human MYBPC3 cDNA and introduced to pCDH-CMV-MCS-EF1-copGFP-T2-Puro vector. Lentivirus was generated with the Lipofectamine^{TM} 3000 (Invitrogen) using the psPAX2 and pMD2G packaging vector, and amplified in human embryonic kidney 293T (HEK293T) cells. Multiplicity of infection (MOI) for iPSC-CMs transduction was optimized to levels at which green fluorescent protein (GFP) could be detected in 80-90% cells.

Immunofluorescent staining

Cells were fixed with 4% PFA for 15 minutes, permeabilized with 0.1% Triton X (Sangon Biotech) for 5 minutes, and blocked with 3% BSA (Sigma-Aldrich) for 1 hour. Cells were subsequently stained with appropriate primary antibodies and

AlexaFluor conjugated secondary antibodies. Primary antibodies include NANOG (Santa Cruz Biotechnology, sc-33759, 1:200), SOX2 (Abcam, ab97959, 1 μg/ml), TNNT2 (Abcam, ab45932, 1:400), α-actinin (Abcam, ab137346, 1:500), atrial natriuretic factor (ANF) (Santa Cruz Biotechnology, sc-20158, 1:200), MYBPC3 (Santa Cruz Biotechnology, sc-137180, 1:200) and nuclear factor of activated T cells (NFAT3) (Affinity, DF-8682, 1:200). Secondary antibodies include AlexaFluor® 647 (Abcam, ab150079, 1:500), AlexaFluor® 488 (Life Technologies, A-11209, 1 μg/ml) and AlexaFluor® 488 (Abcam, ab150129, 1:500). Nuclei were stained with DAPI (Roche Diagnostics, 1023276001, 1 μg/ml). Pictures were taken with 60× objective on confocal microscope (Nikon, A1) using NIS-Elements AR software (Nikon).

Ca^{2+} imaging

The iPSC-CMs grown on coverslips were loaded with RPMI 1640 medium without Phenol Red (Invitrogen) supplemented with 5 μM Fura-2 AM (the stock of Fura-2 AM was pre-dissolved in 20% Pluronic F-127 solution in dimethyl sulfoxide (DMSO)) for 30 minutes in the dark at room temperature. After washing with pre-warmed DPBS and RPMI 1640 for two times, before experiment the cells were immersed in imaging buffer for 30 minutes. Fluorescent signals obtained upon excitation at 340 nm (F340) and 380 nm (F380). Amplitude of Ca^{2+} transient is defined as the ratio of F340/F380.

Assessment of ryanodine receptor 2 (RYR2)-mediated Ca^{2+} leak and sarcoplasmic reticulum (SR) Ca^{2+} load

RYR2-mediated diastolic Ca^{2+} leak and cardiomyocyte SR Ca^{2+} load was assayed

using fluo-4 fluorescence. Cardiomyocytes of day 25-30 were incubated for 15 minutes at room temperature in phenol red-free RPMI 1640 containing 5 μM of the cytosolic Ca^{2+} dye fluo-4 AM (ThermoFisher Scientific) to load the indicator into the cytosol. Following incubation, the indicator-containing medium was removed; cells were washed with phenol red-free RPMI 1640 three times, and immersed in imaging buffer for an additional 30-45 minutes at room temperature to allow for de-esterification of the indicator. Recordings were captured using an epifluorescence microscope. Cells were bathed in 37°C normal Tyrode's (NT) solution (140 mM NaCl, 4 mM KCl, 1.0 mM $MgCl_2$, 5 mM HEPES, 2 mM $CaCl_2$, 10 mM glucose, pH 7.4 with NaOH) for 15-20 seconds and the bathing superfusate was rapidly switched to a 0 Na^+, 0 Ca^{2+} Tyrode buffer (140 mM LiCl, 4 mM KCl, 1 mM $MgCl_2$, 5 mM HEPES, 10 mM glucose, 10 mM EGTA, pH 7.4 with LiOH) for 10 seconds to abolish transsarcolemmal Ca^{2+} fluxes through the Na^+/Ca^{2+} exchanger (NCX). Tetracaine and caffeine solutions were added dropwise using a multi-channel perfusion system (RSC-200, BioLogic). RYR2 channels were inhibited by adding a 1 mM tetracaine (0 Na^+, 0 Ca^{2+}) solution dropwise for ~15 seconds. At this time, tetracaine was stopped, and SR Ca^{2+} stores were depleted by adding a 10 mM caffeine (0 Na^+, 0 Ca^{2+}) solution dropwise for ~10 seconds. At this time, caffeine was stopped, and the bathing solution was rapidly switched from 0 Na^+, 0 Ca^{2+} Tyrode buffer back to NT solution. Data was acquired using NIS-Elements software (Nikon Instruments Inc). When cells were bathed in NT solution, the action potential-induced Ca^{2+} transient amplitude was defined as $\Delta F_{trans}/F_{0,\ diastolic}$, where ΔF_{trans} is the change in signal between the peak

fluorescence and the minimum diastolic fluorescence ($F_{0,\ diastolic}$). The tetracaine-induced drop in fluorescence was defined as $\Delta F_{tet}/F_{0,\ diastolic}$, where ΔF_{tet} is the change in signal between the diastolic fluorescence preceding the addition of tetracaine ($F_{0,\ diastolic}$), and the minimum fluorescence after tetracaine ($F_{0,\ leak}$) under 0 Na^+, 0 Ca^{2+} conditions. The caffeine-induced Ca^{2+} transient amplitude was defined as $\Delta F_{caff}/F_{0,\ diastolic}$, where ΔF_{caff} is the difference between peak fluorescence after caffeine and the minimum diastolic fluorescence. Fractional Ca^{2+} release was defined as the ratio of the action potential-induced Ca^{2+} transient amplitude to the caffeine-induced Ca^{2+} transient amplitude ($\Delta F_{trans}/F_{0,\ diastolic}$)/($\Delta F_{caff}/F_{0,\ diastolic}$).

RNA-Sequencing

After total RNA was extracted, mRNA was isolated by Oligo Magnetic Beads and cut into small fragments for cDNA synthesis. The clustering of the index-coded samples was performed on a cBot Cluster Generation System using TruSeq PE Cluster Kit v3-cBot-HS (Illumia) according to the manufacturer's instructions. And the library preparations were sequenced on an Illumina Novaseq platform. FeatureCounts v1.5.0-p3 was used to count the reads numbers mapped to each gene. Differential expression analysis of WT and L460fs iPSC-CMs was performed using the DESeq2 R package (1.20.0). Genes with an adjusted p value of < 0.05 found by DESeq2 were assigned as differentially expressed.

Quantitative real-time PCR (qPCR)

RNeasy Mini Kit (Qiagen) was applicated for total RNA isolation. RNA concentration was measured using UV spectrophotometry at 260 nm (Nanodrop 2000, Thermo

Scientific). cDNA was obtained using the High Capacity cDNA Reverse transcription Kit (Applied Biosystems). qPCR was performed using SYBR Green PCR Master Mix (Takara). Primer sequences used in this study were listed in **Table S2**. Each reaction was run in quadruplicate using an Applied Biosystems Viia7 Dx (ThermoFisher Scientific). Gene expression values were normalized to the average expression of housekeeping gene GAPDH.

Cycloheximide treatment

The iPSC-CMs were treated with cycloheximide (CHX) for 6 hours before cells were lysed with lysis buffer (150 mM NaCl, 20 mM Tris, 1% Triton X-100, 1% sodium deoxycholate, 0.1% SDS, 10 mM EDTA, and proteinase inhibitor mixture (Roche Applied Science), pH 8.0) at 4°C for 30 minutes. The cell lysates were centrifuged at 13,000 g for 10 minutes to yield protein extracts in the supernatant. The supernatants were then subjected to Western blot analysis.

Western blot

The iPSC-CMs were grown in 6-well plates to 80% confluence, detached with TrypLE (Gibco), and then pelleted at 1000 rpm for 5 minutes at 4°C. After washing with phosphate buffered saline (PBS) (Sangon Biotech), the pellets were re-suspended in 50-100 μl lysis buffer. Lysates were placed on ice for 30 minutes and then the supernatants were collected after centrifuging at 12000 rpm for 15 minutes. Protein concentration was measured using a BCA kit (ThermoFisher Scientific). Western blot was performed using standard protocol with the following antibodies: MYBPC3 (Santa Cruz Biotechnology, sc-137180, 1:200), ANF (Santa Cruz Biotechnology,

sc-515701, 1:100), BNF (Santa Cruz Biotechnology, sc-271185, 1:100), HSC70 (Abcam, ab51052, 1:800) and GAPDH (Abmart, M200006, 1:1000).

Compounds and solutions

Fura-2 AM and Fluo-4 AM were purchased from Thermo Fisher Scientific and stock solutions were both prepared in 1 mM in 20% Pluronic F-127 (Sangon Biotech) dissolved in DMSO. Ang II was purchased from Sigma-Aldrich and stock solutions were prepared in 10 mM in water. VER-155008 was purchased from MCE and stock solutions were prepared in 10 mM in DMSO. YM-1 was purchased from Sigma-Aldrich and stock solutions were prepared in 10 mM in DMSO. CHX was purchased from Selleck and stock solutions were prepared in 10 mg/ml in water. MG132 and chloroquine (CQ) were both purchased from Targetmol and stock solutions were prepared in 10 mM and 20 mM in DMSO, respectively.

Data availability

The accession number for the RNA-Seq data reported in this study is NCBI SRA: PRJNA746712.

Statistical analysis

Statistical significance was determined by unpaired two-tailed Student's t-test to compare two groups and by One-way ANOVA to compare multiple groups. A *p* value of < 0.05 was considered statistical significance. Data were shown as Mean ± SD and analyzed by GraphPad Prism 8 (GraphPad Software).

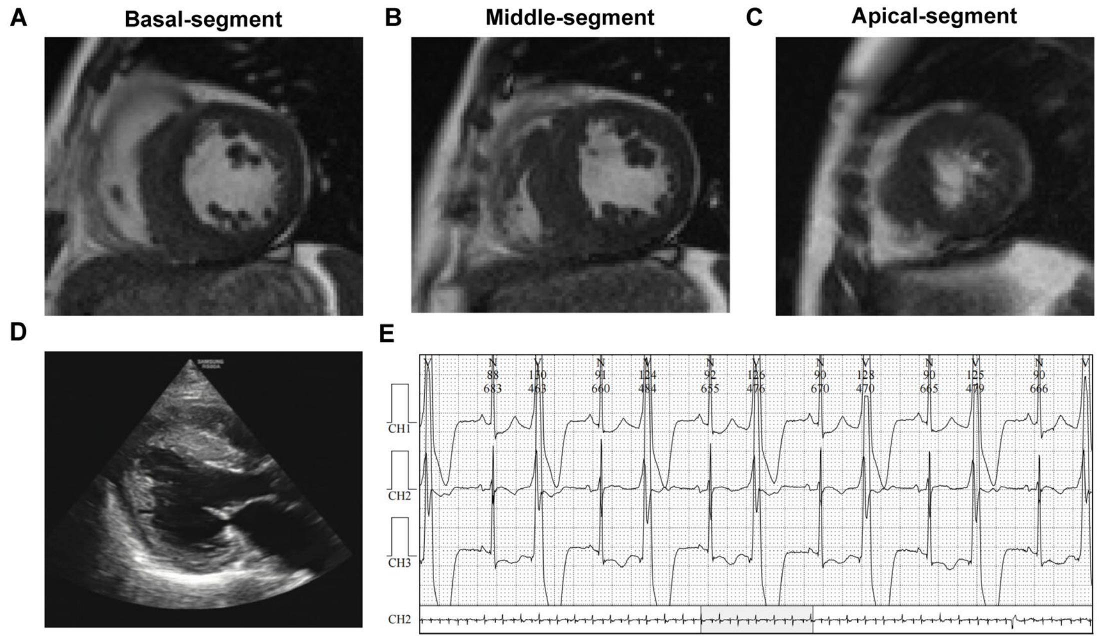

Figure S1. Morphological and ECG characteristics of probands. A-C. Representative short-axis MRI images of proband 4 at end diastole from basal to apical segment demonstrating basal and middle septal hypertrophy. **D.** Representative long-axis echocardiography of proband 4 at end diastole with asymmetric hypertrophy of the septum and elongated anterior mitral valve. **E.** 24-hour Holter image presenting a dual law of premature ventricular contraction in proband 2.

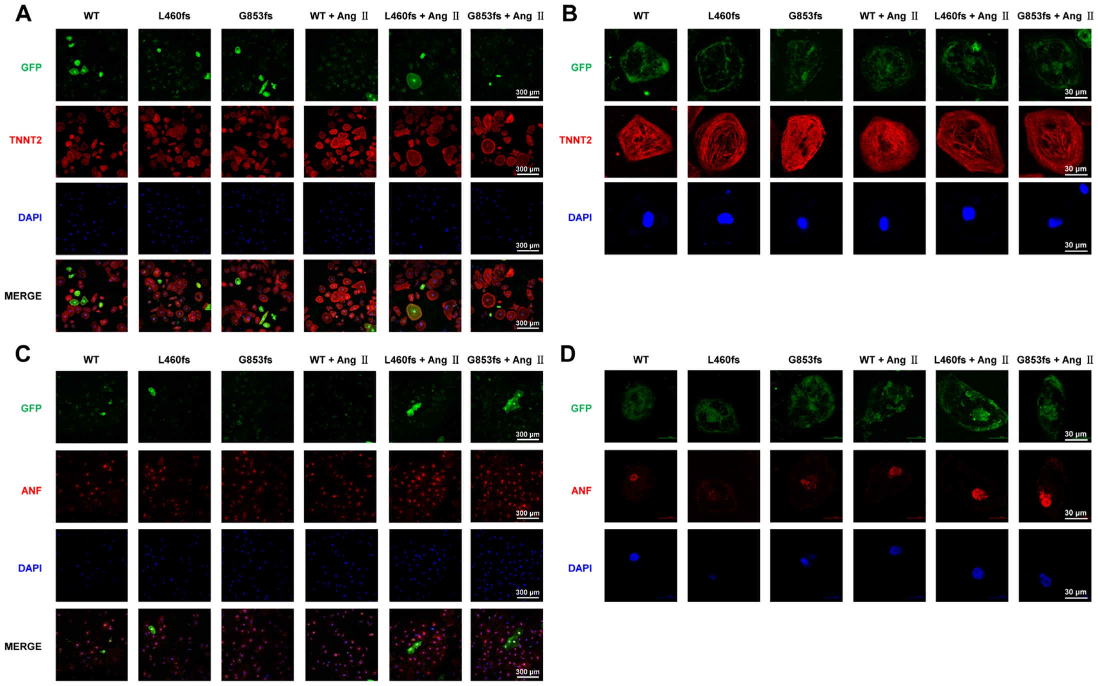

Figure S2. Enlarged cell size and upregulated *ANF* expression in Ang II-treated iPSC-CMs overexpressing MYBPC3 truncation mutations. A-B. Representative graphs of cardiac-specific staining of GFP (green) and TNNT2 (red) in basal WT iPSC-CMs (WT Basal), Ang II-treated WT iPSC-CMs (WT Ang II), basal mutant iPSC-CMs (L460fs or G853fs Basal) and Ang II-treated mutant iPSC-CMs (L460fs or G853fs Ang II) at low (A, scale bar= 300 μm) and high (B, scale bar= 30 μm) magnification, respectively. DAPI indicates nuclear staining (blue). **C-D.** Representative graphs of staining of GFP (green) and hypertrophic-specific marker ANF (red) in different groups at low (C, scale bar= 300 μm) and high (D, scale bar= 30 μm) magnification, respectively. DAPI indicates nuclear staining (blue).

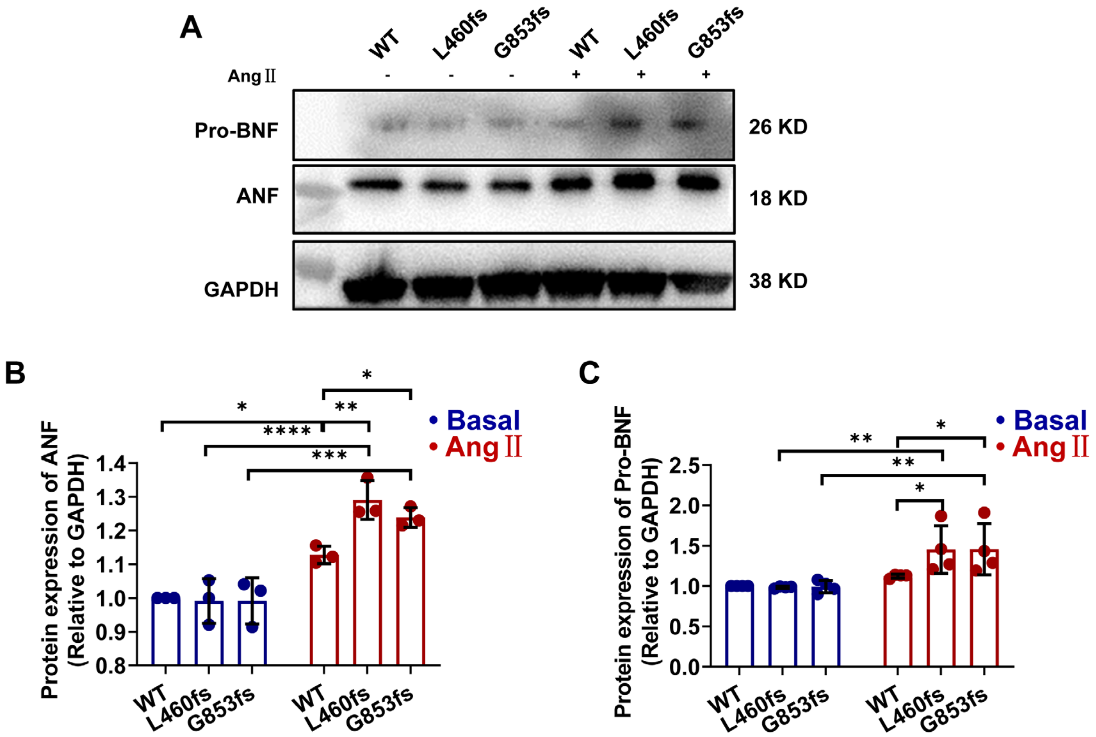

Figure S3. Upregulated protein expression of hypertrophic markers in Ang II-treated mutant iPSC-CMs. A. Western blot analysis of protein expression of ANF and Pro-BNF in WT and mutant iPSC-CMs with or without Ang II treatment. GAPDH is used for the loading control. **B-C.** Bar graphs to compare the protein expression of ANF and Pro-BNF between different groups. n= 3-4 culture replicates. $^{*}P< 0.05$, $^{**}P< 0.01$, $^{***}P< 0.001$ and $^{****}P< 0.0001$.

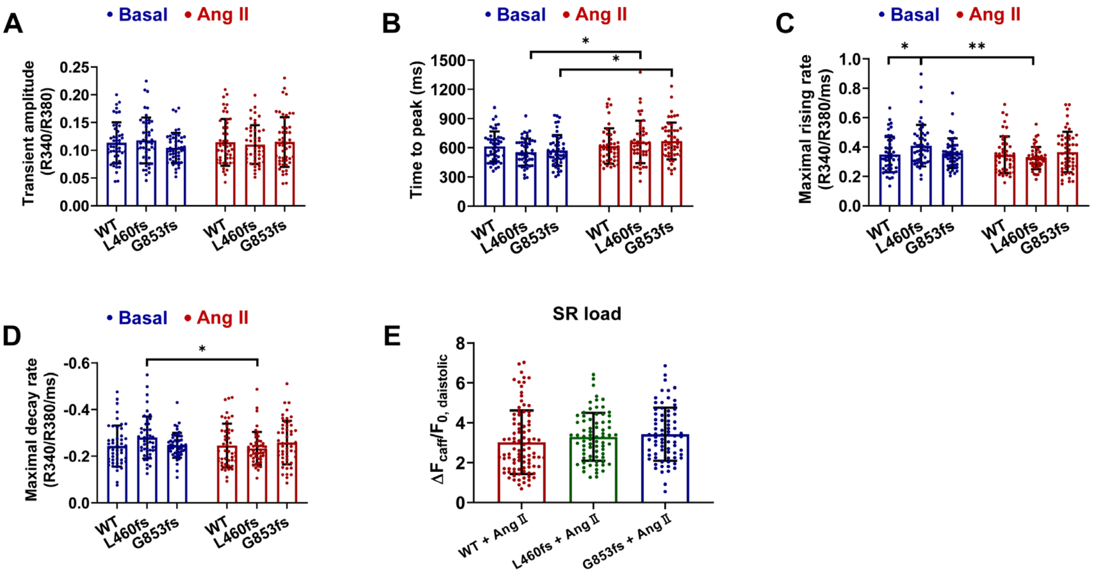

Figure S4. Ang II induction recapitulates Ca^{2+} handling abnormalities in iPSC-CMs overexpressing truncation mutations. A-D. Bar graphs to compare the transient amplitude, time to peak, maximal rising rate, and maximal decay rate between different groups. n= 47-51 cells. $^{*}P< 0.05$ and $^{**}P< 0.01$. **E.** Bar graphs to compare the SR Ca^{2+} load between different groups. n= 74-93 cells.

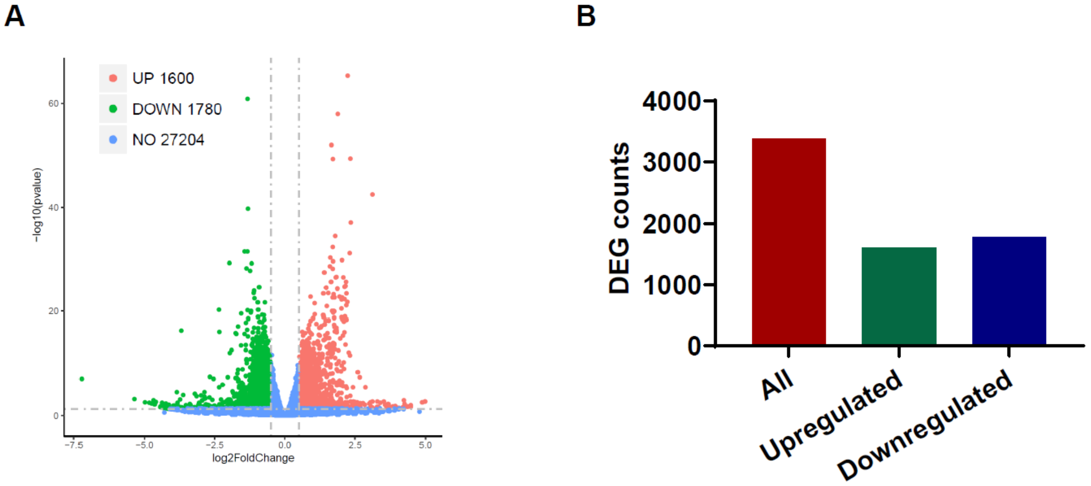

Figure S5. RNA-Seq of MYBPC3 L460fs iPSC-CMs reveals a common gene signature of HCM and upregulated HSC70 expression. A. Volcano plot of differential expressed genes (DEGs). Red points represent upregulated DEGs. Green points represent downregulated DEGs. Blue points represent non-DEGs. We observed that 3380 genes out of 30584 total genes were differentially expressed in Ang II-treated L460fs iPSC-CMs when compared to WT, in which 1600 DEGs were upregulated and 1780 DEGs were downregulated**. B.** Bar graph to demonstrate the number of all, upregulated and downregulated DEGs.

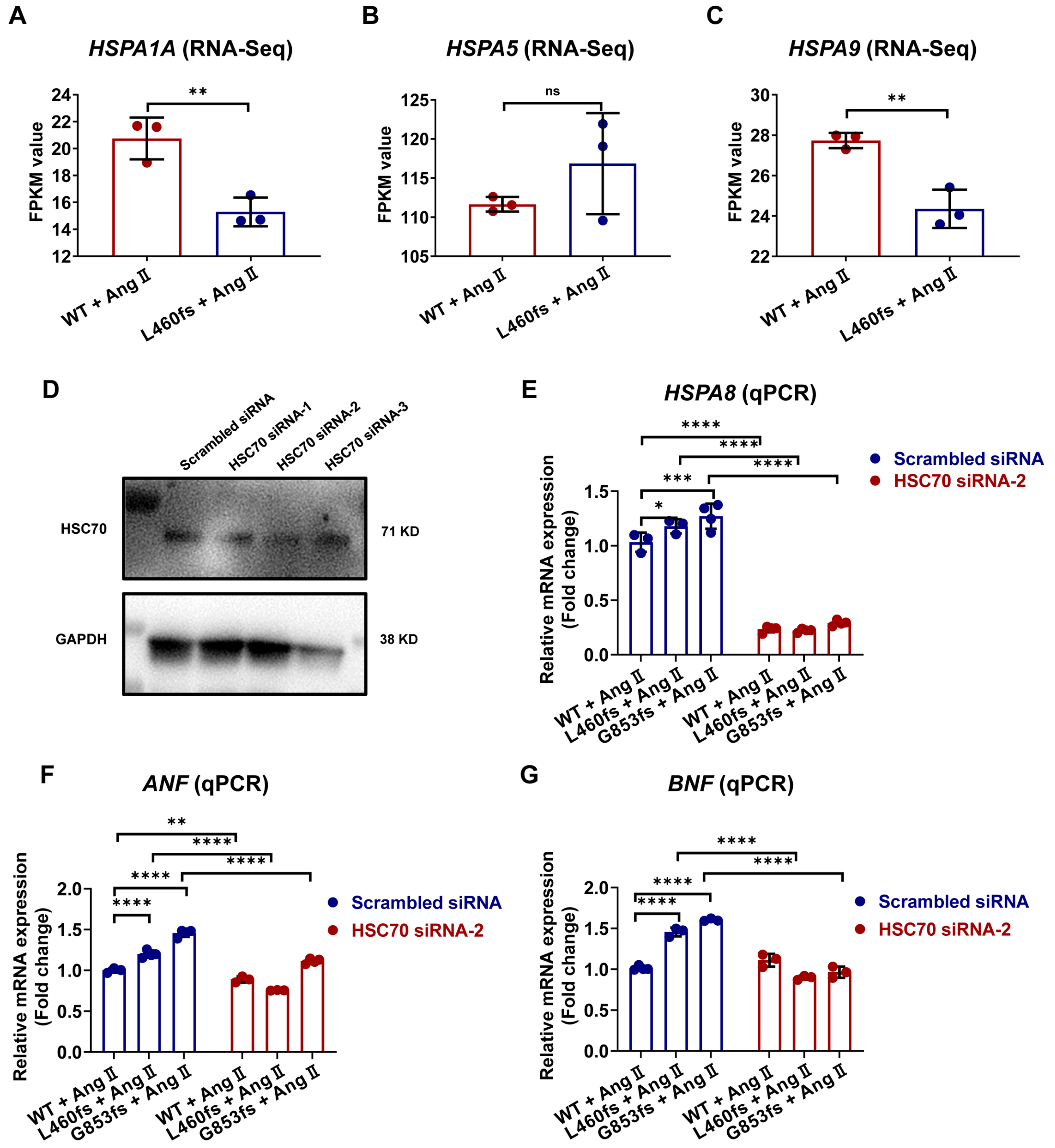

Figure S6. Knockdown of HSC70 suppressed Ang II-induced hypertrophy. A-C. Bar graphs to compare the mRNA expression of *HSPA1A*, *HSPA5* and *HSPA9* between Ang II-treated WT and L460fs iPSC-CMs by RNA-Seq. n= 3 technical replicates. $^{*}P< 0.05$, $^{**}P< 0.01$ and $^{***}P< 0.001$. **D.** Western blot analysis of HSC70 expression in healthy iPSC-CMs after 48-hour HSC70 siRNA treatment. Three siRNAs (siRNA-1, siRNA-2 and siRNA-3) were tested and siRNA-2 was selected for knockdown of HSC70 in further functional investigations. GAPDH is used for the loading control. **E-G.** Bar graphs to compare the mRNA expression of *HSPA8*, *ANF* and *BNF* by qPCR between Ang II-treated WT and mutant iPSC-CMs with scrambled siRNA or HSC70 siRNA, respectively. n= 3-4 technical replicates. $^{*}P< 0.05$, $^{**}P< 0.01$, $^{***}P< 0.001$ and $^{****}P< 0.0001$.

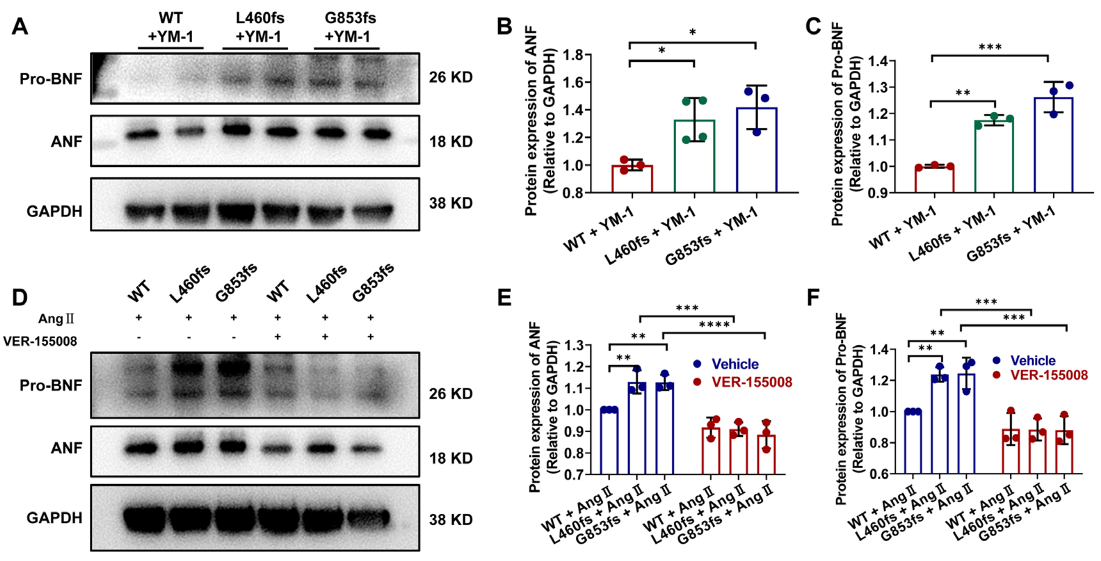

Figure S7. Pharmacological activation or inhibition of HSC70 exacerbated or rescued hypertrophy phenotype. A. Western blot analysis of protein expression of ANF and Pro-BNF in WT and mutant iPSC-CMs after YM-1 treatment. **B-C.** Bar graphs to compare the protein expression of ANF and Pro-BNF between WT and mutant iPSC-CMs after YM-1 treatment. n= 3-4 culture replicates. $^{*}P< 0.05$, $^{**}P< 0.01$ and $^{***}P< 0.001$. **D.** Western blot analysis of protein expression of ANF and Pro-BNF in Ang II-treated WT and mutant iPSC-CMs with or without VER-155008 treatment. **E-F.** Bar graphs to compare the protein expression of ANF and Pro-BNF between Ang II-treated WT and mutant iPSC-CMs with or without VER-155008 treatment. n= 3 culture replicates. $^{**}P< 0.01$, $^{***}P< 0.001$ and $^{****}P< 0.0001$.

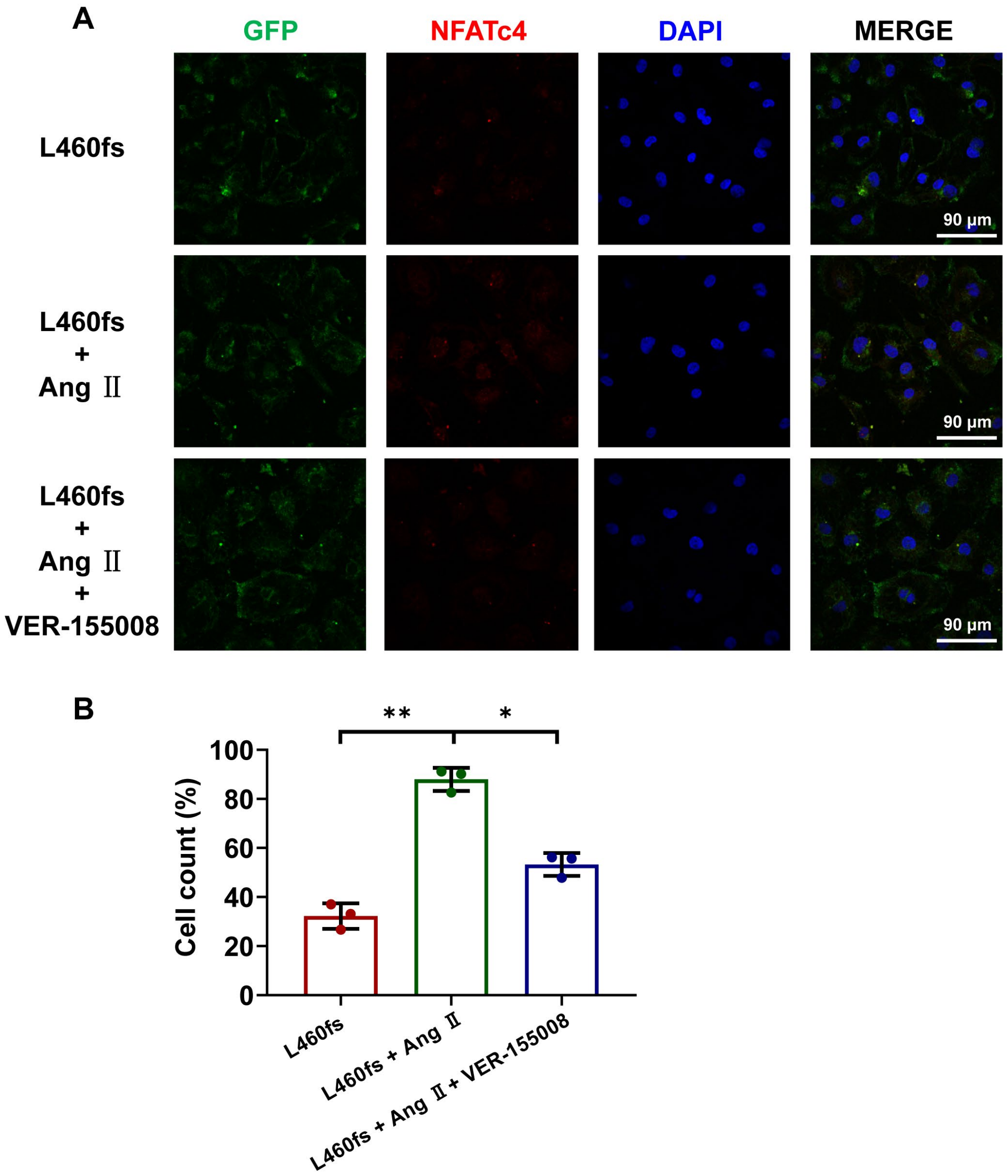

Figure S8. Inhibition of HSC70 activity suppressed Ang II-induced translocation of NFATc4. A. Representative graphs of staining of GFP (green) and NFATc4 (red) in L460fs iPSC-CMs, Ang II-treated L460fs iPSC-CMs, and iPSC-CMs treated with Ang II and VER-155008, respectively. DAPI indicates nuclear staining (blue). Scale bar, 90 μm. **B.** Bar graph to compare the cell count (%) exhibiting nuclear translocation of NFATc4 in different groups. n= 246-387 cells. $^{*}P< 0.05$ and $^{**}P< 0.01$.

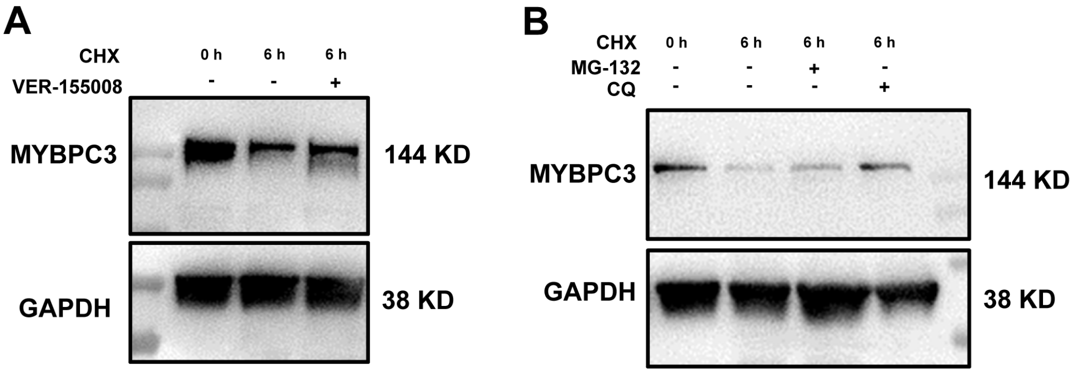

Figure S9. HSC70 participates in MYBPC3 protein degradation via lysosomal pathway in iPSC-CMs. A. Western blot analysis of MYBPC3 expression in healthy iPSC-CMs after 6-hour treatment of CHX (120 μg/ml, 6 hours) with or without VER-155008. **B.** Western blot analysis of MYBPC3 expression in healthy iPSC-CMs after 6-hour treatment of CHX with MG-132 (30 μM, 6 hours) or CQ (50 μM, 6 hours).

Table S1. Clinical features of four recruited probands

Patient ID	Proband 1	Proband 2	Proband 3	Proband 4
**Age (years)**	59	56	41	57
**MYBPC3 genotype**	p.L460fs	p.L460fs	p.L460fs	p.L460fs
**Heart rate (bpm)**	72	79	65	55
**QT (ms)**	410	407	416	414
**SBP (mm Hg)**	125	127	126	117
**DBP (mm Hg)**	64	78	76	74
**LVEDD (mm)**	49	44	35	52.4
**LVESD (mm)**	33	24	19	37.8
**LVEDV (ml)**	111	88	52	132
**LVESV (ml)**	44	20	12	61.2
**LVPWD (ml)**	10	19.3	12.1	11.9
**IVSDD (mm)**	25.1	20.9	20.5	24.7
**LVEF (%)**	60.1	76	77.4	59.7
**LVOT Gr (mm Hg)**	3.9	0	16	5
**LAD (mm)**	44	35.4	41	41.8
**Holter monitor**	paroxysmal atrial fibrillation	Occasional PVCs	Occasional PVCs	non-sustained VT
**Symptoms**	Exertional chest pain, palpitations	Exertional chest pain, palpitations	Palpitations	Exertional chest pain, palpitations, dizziness

Table S2. Primers used for qPCR in this study

Genes	Primer sequences	
ANF	Forward	CAGGATGGACAGGATTGGA
	Reverse	TGTCCTCCCTGGCTGTTATC
BNF	Forward	TCAGCCTGGACTTGGAAAC
	Reverse	CTTCCAGACACCTGTGGGAC
MYBPC3	Forward	CAAGGTCTATCTGTTCGAGCTG
	Reverse	AGAATCCCAGTGTCCTCATGG
HSPA8	Forward	ACTCCAAGCTATGTCGCCTTT
	Reverse	TGGCATCAAAAACTGTGTTGGT
GAPDH	Forward	GGTCGGAGTCAACGGATTTG
	Reverse	CGGTGCCATGGAATTTGCC

Table S3. Summary of key parameters of Ca^{2+} transients in WT and mutant iPSC-CMs

	Diastolic (F340/F380)	Transient amplitude (F340/F380)	Time to peak (ms)	Maximal rising rate (F340/F380/s)	Maximal decay rate (F340/F380/s)
**WT basal**	0.108 ± 0.027	0.113 ± 0.037	612.7 ± 154.8	0.349 ± 0.121	-0.243 ± 0.088
**L460fs basal**	0.116 ± 0.029	0.118 ± 0.041	555.3 ± 138.9	0.409 ± 0.142	-0.280 ± 0.090
**G853fs basal**	0.112 ± 0.039	0.104 ± 0.027	572.1 ± 158.6	0.357 ± 0.101	-0.246 ± 0.053
**WT Ang II**	0.125 ± 0.027	0.114 ± 0.042	617.4 ± 182.9	0.347 ± 0.124	-0.245 ± 0.094
**L460fs Ang II**	0.143 ± 0.027	0.110 ± 0.035	659.0 ± 217.6	0.325 ± 0.076	-0.233 ± 0.071
**G853fs Ang II**	0.145 ± 0.042	0.115 ± 0.045	668.4 ± 189.3	0.365 ± 0.138	-0.258 ± 0.094

Table S4. Summary of diastolic $[Ca^{2+}]_i$, RYR2-mediated Ca^{2+} leak and SR Ca^{2+} load in WT and mutant iPSC-CMs

	Diastolic $[Ca^{2+}]_i$	RYR2-mediated Ca^{2+} leak	SR Ca^{2+} load
**WT Ang II**	11.022 ± 5.061	0.279 ± 0.097	3.025 ± 1.594
**L460fs Ang II**	13.889 ± 7.657	0.342 ± 0.107	3.294 ± 1.205
**G853fs Ang II**	13.414 ± 6.599	0.350 ± 0.101	3.426 ± 1.333
**WT Ang II + VER**	10.552 ± 4.267	0.303 ± 0.075	2.932 ± 1.179
**L460fs Ang II + VER**	10.310 ± 4.455	0.282 ± 0.087	2.742 ± 1.162
**G853fs Ang II + VER**	10.055 ± 3.067	0.289 ± 0.067	2.603 ± 0.832

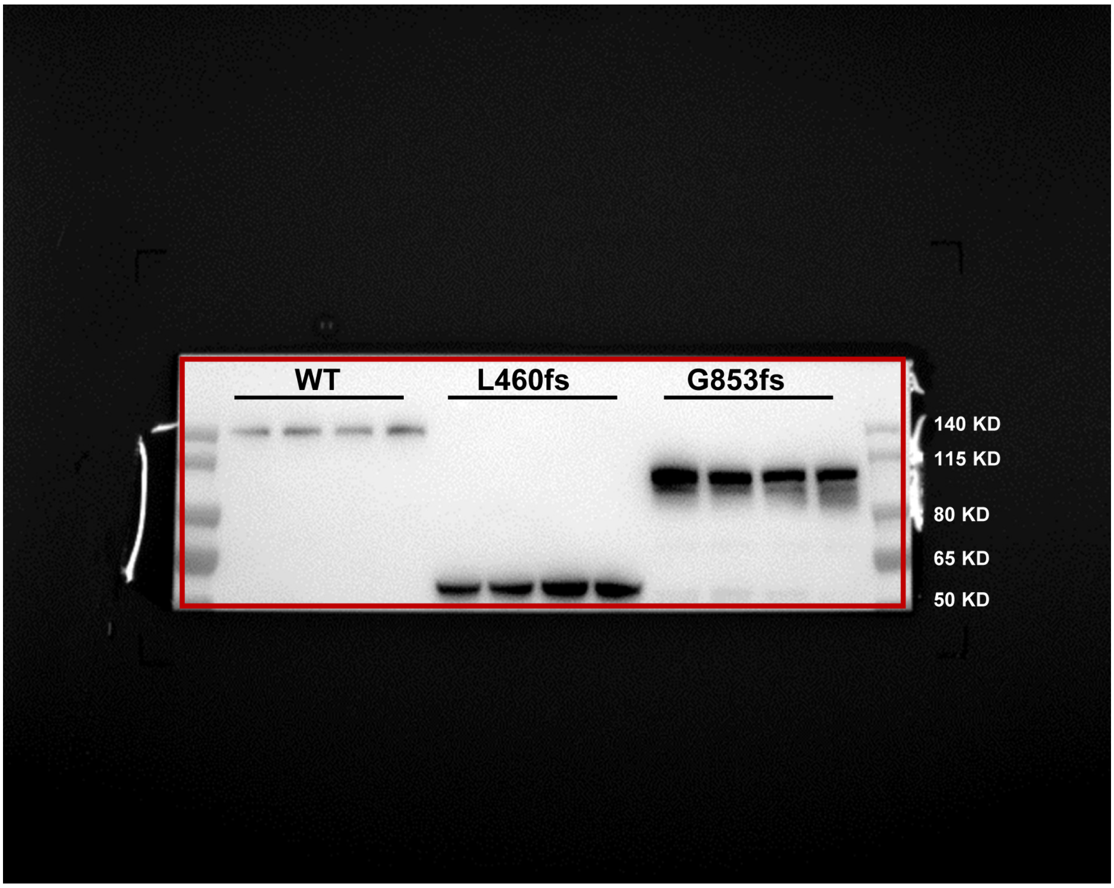

Full length blots of WT and truncated (p.L460fs, p.G853fs) MYBPC3 overexpressed in HEK293T cells. Red boxes indicate the cropped blots shown in Figure 1I.

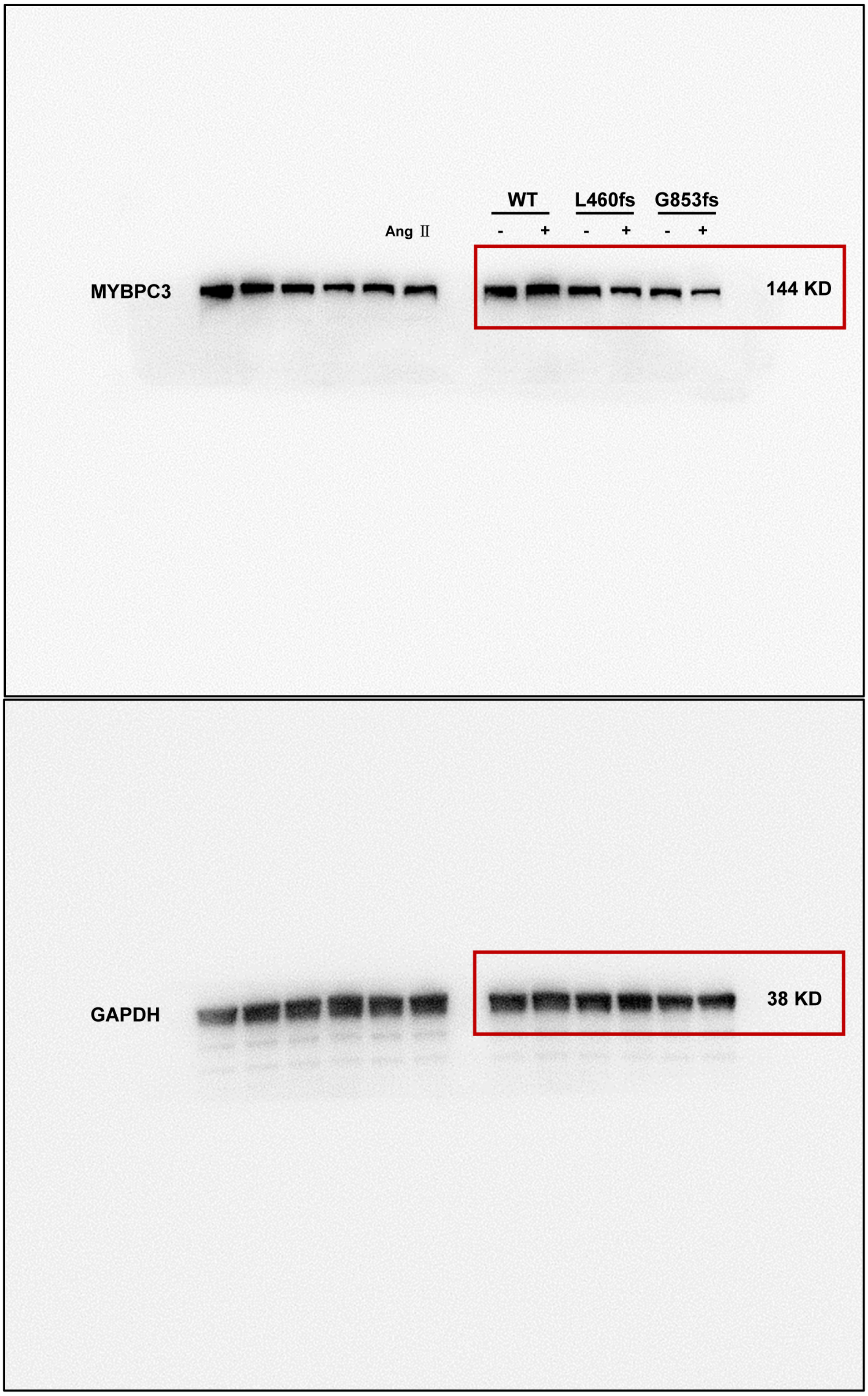

Full length blots of MYBPC3 expression in WT and mutant iPSC-CMs with or without Ang II treatment. Red boxes indicate the cropped blots shown in Figure 2G.

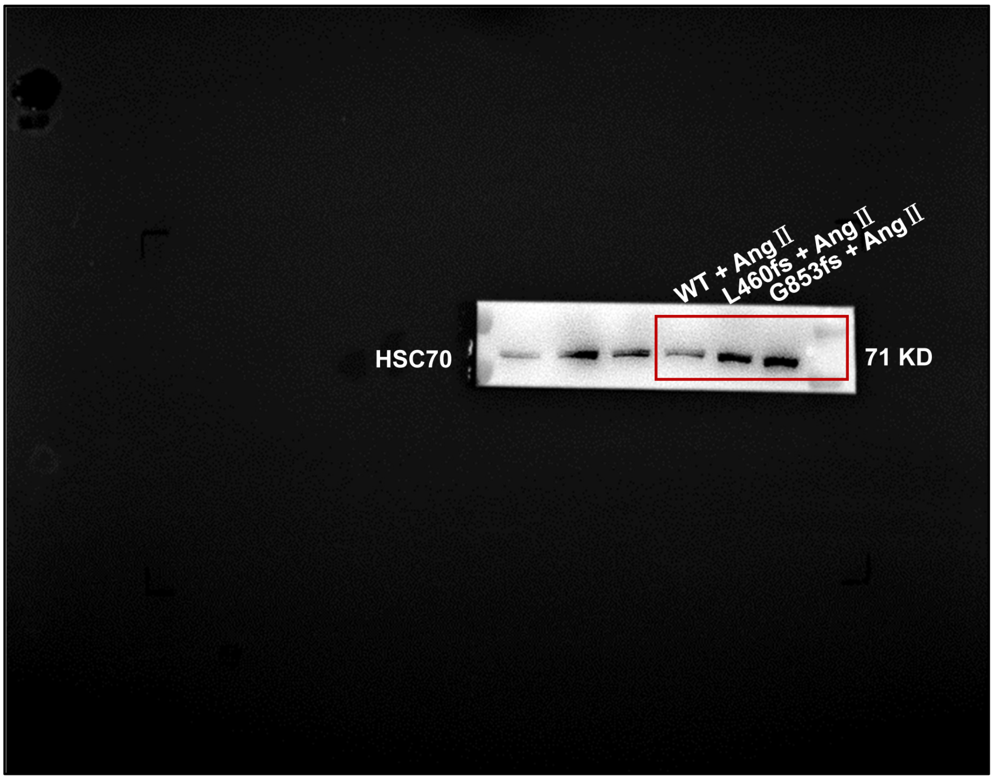

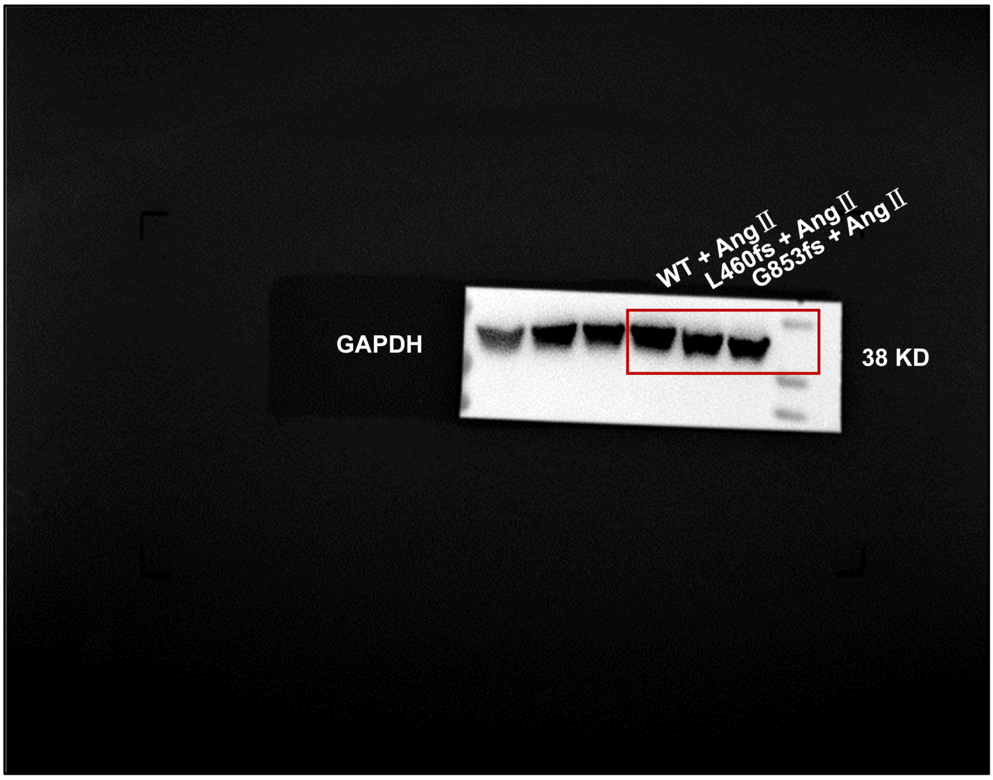

Full length blots of HSC70 expression in WT and L460fs iPSC-CMs after Ang II treatment. Red boxes indicate the cropped blots shown in Figure 3F.

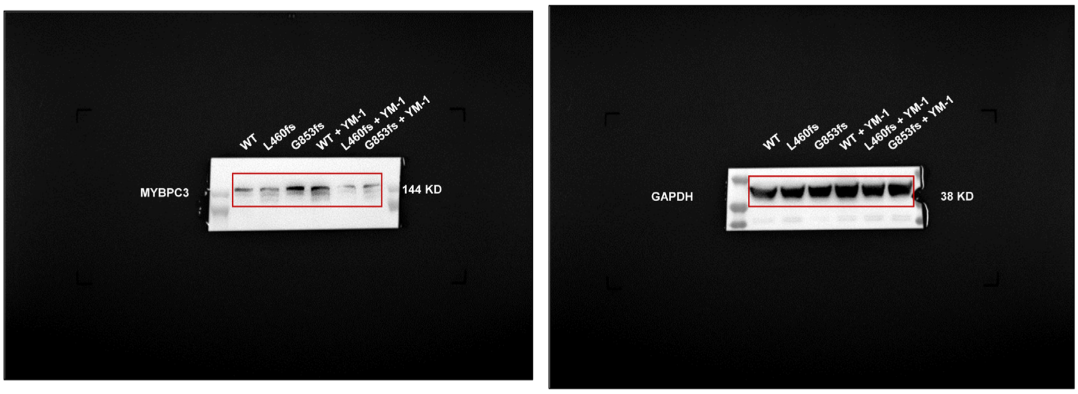

Full length blots of MYBPC3 expression in WT and mutant iPSC-CMs after YM-1 treatment. Red boxes indicate the cropped blots shown in Figure 4A.

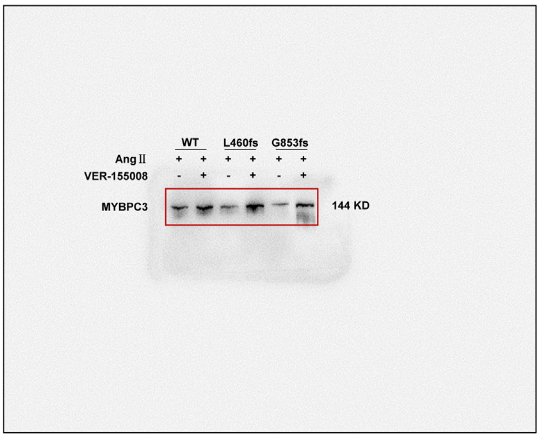

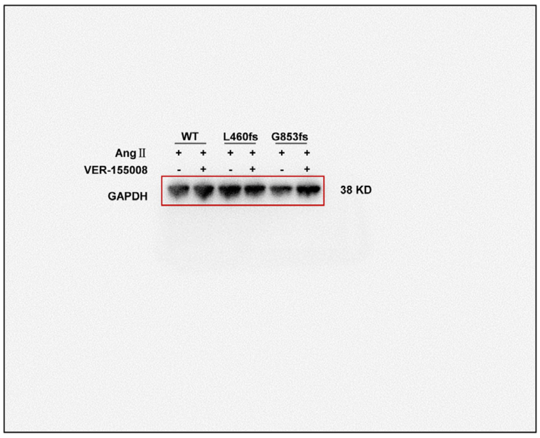

Full length blots of MYBPC3 expression in WT and mutant iPSC-CMs after VER-155008 treatment. Red boxes indicate the cropped blots shown in Figure 4C.

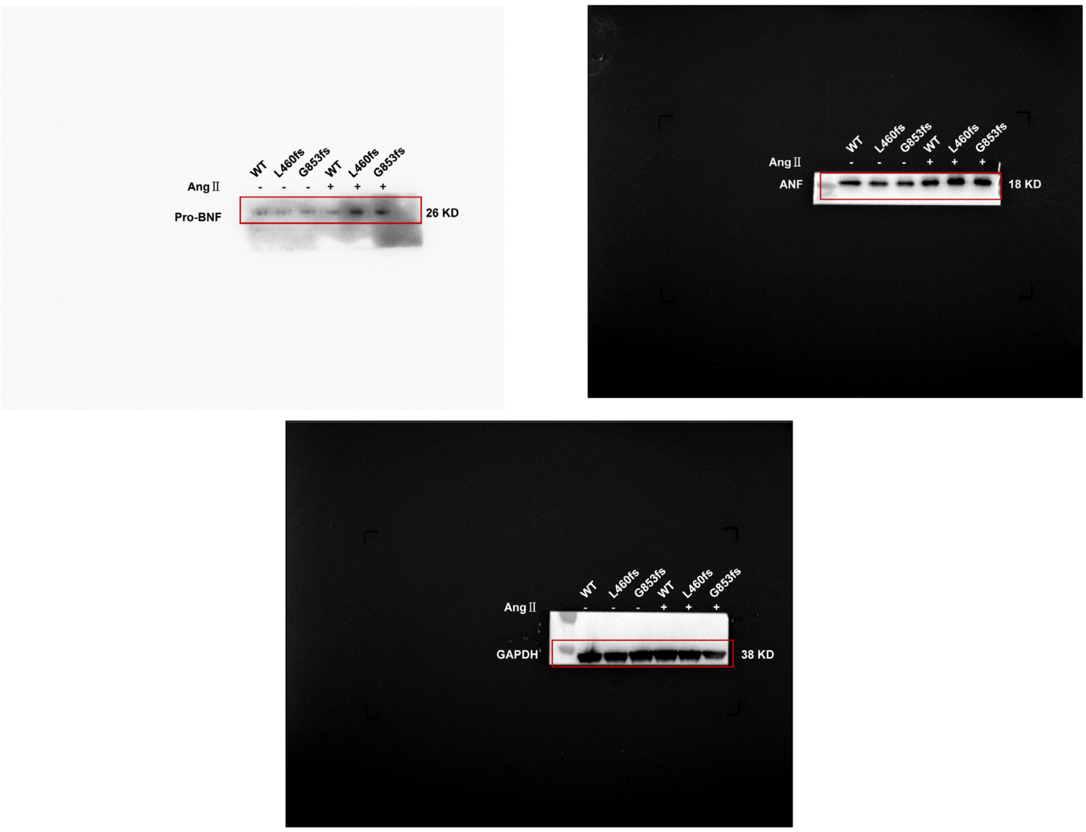

Full length blots of protein expression of ANF and Pro-BNF in WT and mutant iPSC-CMs with or without Ang II treatment. Red boxes indicate the cropped blots shown in Figure S3A.

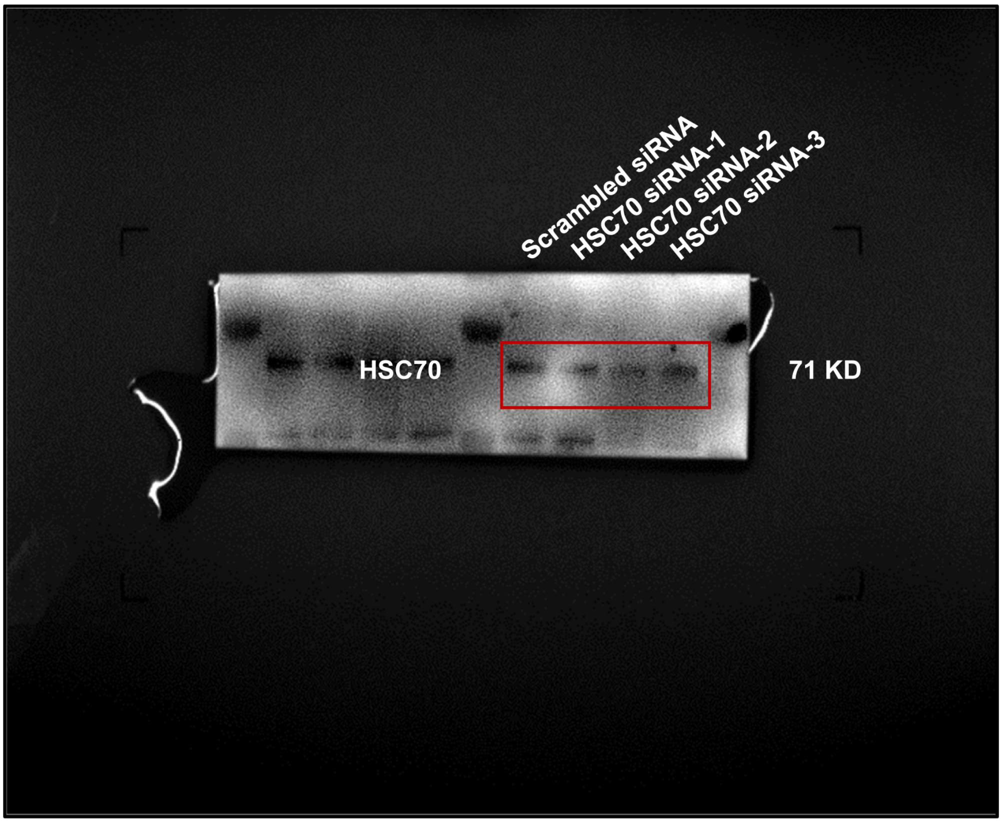

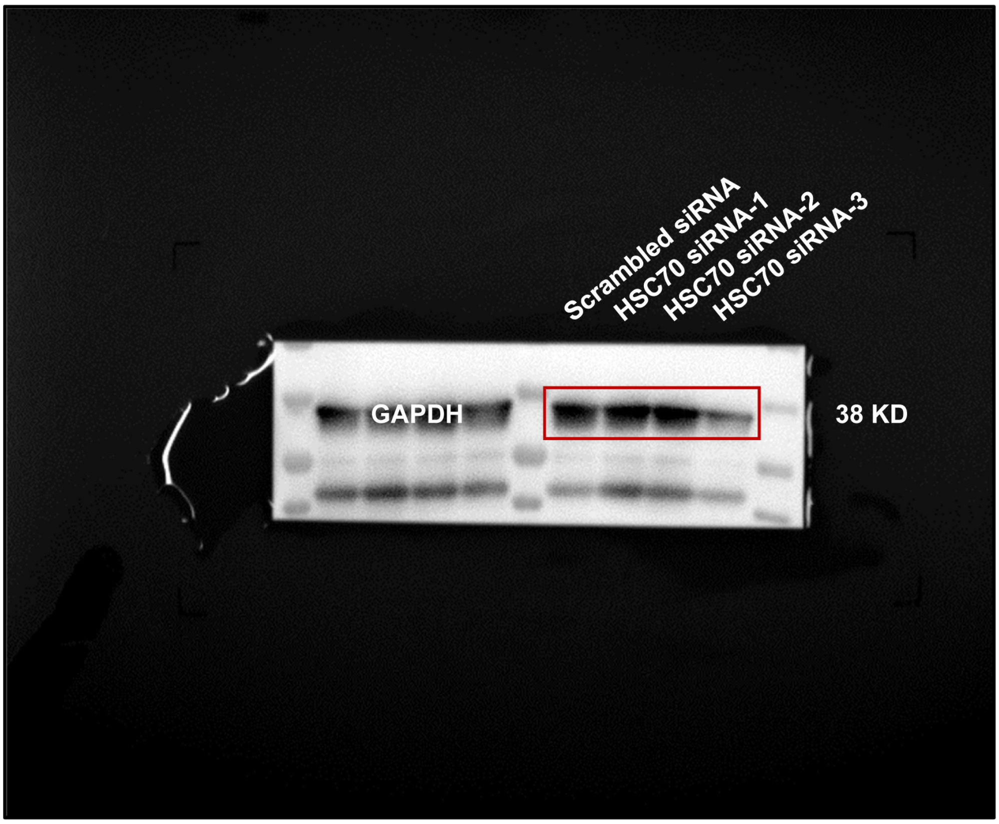

Full length blots of HSC70 expression in healthy iPSC-CMs after 48-hour HSC70 siRNA treatment. Red boxes indicate the cropped blots shown in Figure S6D.

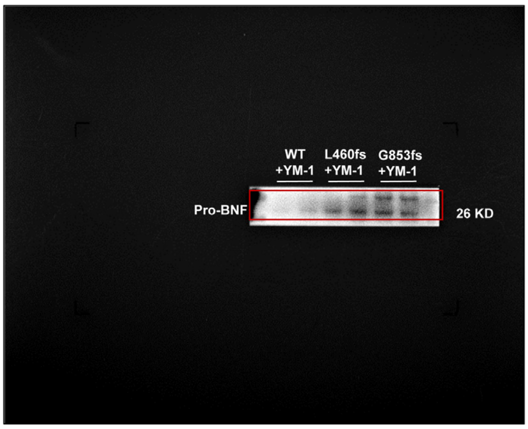

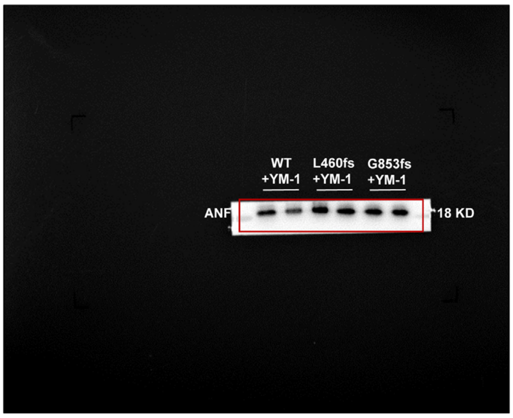

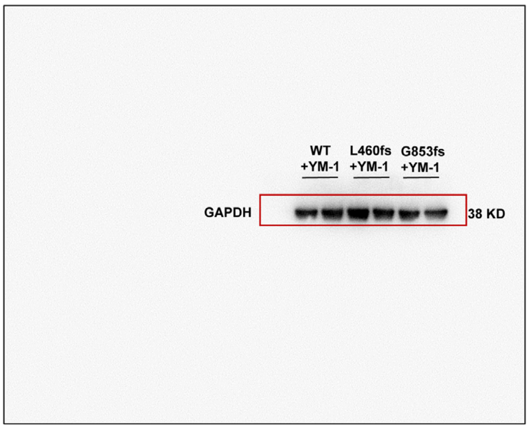

Full length blots of protein expression of ANF and Pro-BNF in WT and mutant iPSC-CMs after YM-1 treatment. Red boxes indicate the cropped blots shown in Figure S7A.

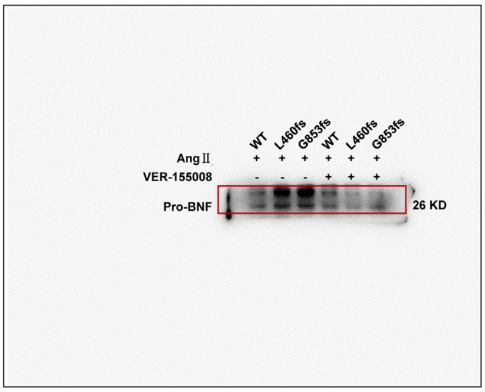

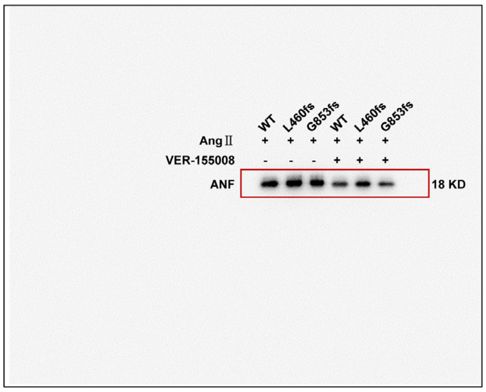

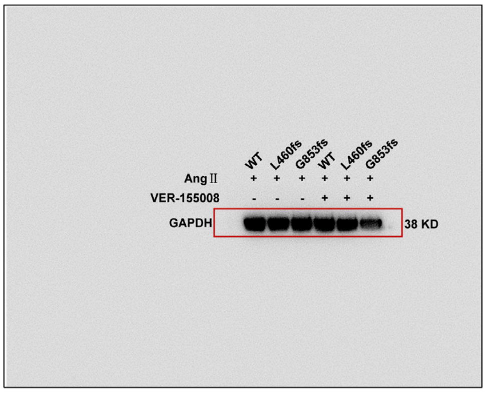

Full length blots of protein expression of ANF and Pro-BNF in Ang II-treated WT and mutant iPSC-CMs with or without VER-155008 treatment. Red boxes indicate the cropped blots shown in Figure S7D.

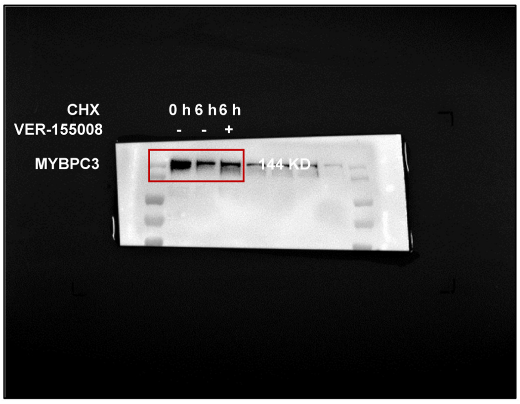

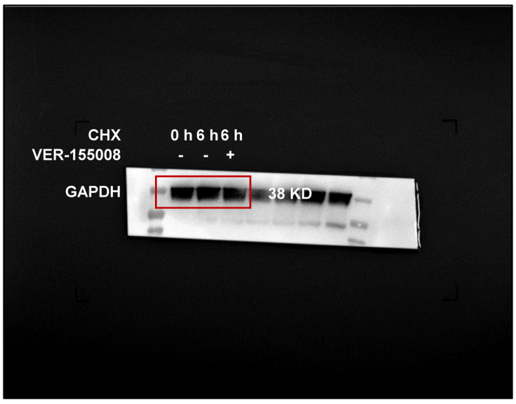

Full length blots of MYBPC3 expression in healthy iPSC-CMs after 6-hour treatment of CHX with or without VER-155008. Red boxes indicate the cropped blots shown in Figure S9A.

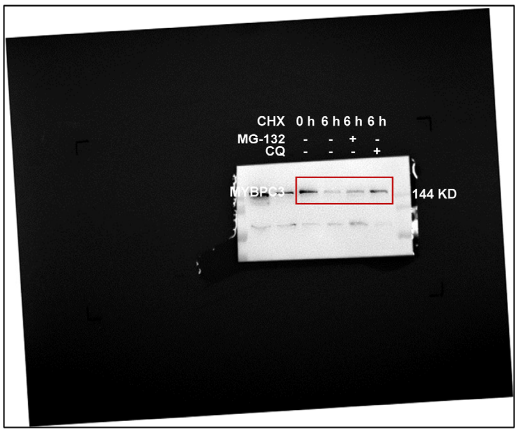

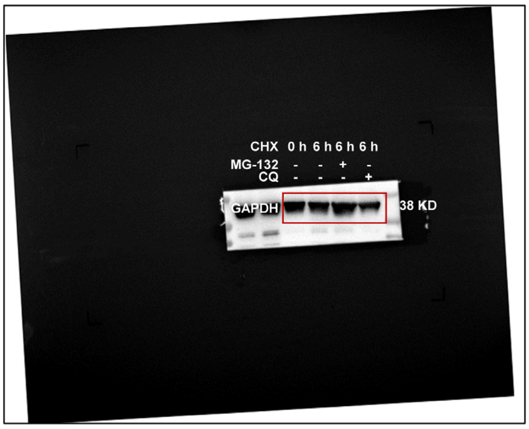

Full length blots of MYBPC3 expression in healthy iPSC-CMs after 6-hour treatment of CHX with MG-132 or CQ. Red boxes indicate the cropped blots shown in Figure S9B.
